# Aryl hydrocarbon receptor deficiency leads to sex- and age-dependent colonic dysmotility in mice

**DOI:** 10.1007/s13105-026-01208-9

**Published:** 2026-07-16

**Authors:** Alicia Valls, David Verdú, Sol Guerra-Ojeda, Teresa San-Miguel, Andrea Suarez, Javier Pereda, Verónica Gómez-Jiménez, José Viña, Maria D Mauricio, Eva Serna

**Affiliations:** 1 Department of Physiology, Facultat de Medicina i Odontologia, Universitat de Valencia, Valencia, 46010 Spain; 2 Department of Pathology, Facultat de Medicina i Odontologia, Universitat de Valencia, Valencia, 46010 Spain; 3 MODULAhR Group, Universitat de Valencia, Valencia, 46010 Spain; 4 Centro de Investigación Biomédica en Red Fragilidad y Envejecimiento Saludable CIBERFES, INCLIVA Biomedical Research Institute, Valencia, 46010 Spain; 5 Centro de Investigación Biomédica en Red Enfermedades Cardiovasculares CIBERCV, INCLIVA Biomedical Research Institute, Valencia, 46010 Spain

**Keywords:** Aryl hydrocarbon receptor, Intestinal motility, Colon, Enteric nervous system, Sex differences, Aging

## Abstract

The aryl hydrocarbon receptor (AhR) is a ligand-activated transcription factor involved in xenobiotic sensing and multiple physiological functions, including intestinal homeostasis. Aging is associated with reduced colonic motility and constipation, but the contribution of AhR to intestinal motor function across sex and age remains incompletely defined. We performed a phenotypic characterization of AhR-deficient (AhR^−/−^) and wild-type (WT) C57BL/6J mice in both sexes at two ages (young: 4–6 months; adult: 12–13 months). Lifespan, body weight, food and water intake were monitored longitudinally. Body composition (DXA), hematological parameters, fecal output features (number, length, moisture and stool color analysis), ex vivo colonic function in organ bath (spontaneous peristalsis with or without L-NAME and responses to acetylcholine and norepinephrine), histological muscle thickness, and molecular markers (PGP9.5 and NOS1 protein; *Nos1*, *Nos2*, *Aqp1/3/4/8*, *Il6*, *Il10* gene expression) were assessed. AhR^−/−^ mice exhibited reduced survival and attenuated body weight gain, particularly in females, without differences in food consumption but with increased water intake. Fecal length and moisture were unchanged; however, AhR^−/−^ females displayed fewer stools at both ages. Ex vivo, spontaneous peristaltic amplitude was selectively reduced in AhR^−/−^ females at both ages, whereas males were unaffected. L-NAME increased spontaneous peristalsis in WT males but not in AhR^−/−^ males, suggesting impaired nitrergic modulation in the absence of AhR. Acetylcholine-induced contraction was reduced in young AhR^−/−^ females but not in adults. Histology revealed reduced colonic muscle thickness in young AhR^−/−^ females only, accompanied by decreased PGP9.5 protein expression. Gene expression showed sex- and age-dependent changes in *Nos1*/*Nos2*, aquaporins, and inflammatory markers. AhR deficiency leads to a sex-dependent colonic motor phenotype predominantly affecting females, integrating functional impairment with early neuromuscular remodeling and altered neural markers. These findings support AhR as a regulator of intestinal motor function with potential relevance to age-related dysmotility.

**Supplementary Information:**

The online version contains supplementary material available at 10.1007/s13105-026-01208-9.

## Introduction

The aryl hydrocarbon receptor (AhR) is a ligand-activated transcription factor originally characterized for its role in xenobiotic sensing and detoxification [1]. Beyond toxicological responses, AhR is now recognized as a pleiotropic regulator involved in immune homeostasis, tissue integrity, metabolism, and aging-related processes [2, 3].

In the gastrointestinal tract, AhR integrates dietary, microbial, and endogenous signals to maintain intestinal homeostasis through coordinated regulation of epithelial barrier function and mucosal immunity [4]. AhR supports the maintenance of innate lymphoid cells (ILCs) and intraepithelial lymphocytes (IELs), promotes interleukin-22 (IL-22) production, and shapes mucosal immune phenotypes in response to microbial and dietary ligands [5, 6]. In parallel, gut microbiota-derived metabolites, particularly those arising from tryptophan catabolism, can serve as endogenous AhR agonists, linking microbial ecology to host metabolism and mucosal function [7]. Several studies suggest that activation of AhR by agonists such as indole-3-carbinol, as well as by tryptophan-derived metabolites generated through bacterial metabolism within the gut microbiota, may improve parameters associated with inflammation and intestinal dysfunction in specific pathological contexts, such as necrotizing enterocolitis, intestinal inflammation, and colitis. These findings underscore the role of AhR as a key receptor in maintaining intestinal homeostasis [8–10].

Aging is accompanied by progressive changes in intestinal physiology, including impaired secretion, digestion and nutrient absorption, and a shift in gut microbial composition [11, 12]. Importantly, colonic motility declines with age, contributing to an increased prevalence of constipation in older populations. Proposed mechanisms include altered neurotransmitter release and reduced neuronal integrity within the enteric nervous system [13–15]. Transcriptomic evidence in humans indicates that the AhR/ARNT signaling pathway undergoes age-dependent modulation, characterized by a decrease in activity in aging individuals and an increase in centenarians [16]. This result suggests that this detoxification response mechanism is maintained in extreme longevity. Furthermore, receptor deficiency has been observed to accelerate the onset of age-related lesions such as premature brain aging [17].

Despite these advances, the specific contribution of AhR to intestinal motor function, particularly across sex and age, remains insufficiently defined. Recent toxicological evidence suggests a potential link between AhR and enteric neuromuscular control specifically, nitrergic neurons [18]. Conversely, whether the absence of AhR leads to intrinsic dysmotility, altered neuromodulatory pathways, or compensatory adaptations is unclear. Moreover, sex as a biological variable may be critical in defining the phenotype, given the known sexual dimorphism in immune regulation, neuromuscular function, and aging trajectories, even more in the capacity response to AhR ligands [19].

Therefore, the aim of this study was to perform a comprehensive phenotypic characterization of AhR^−/−^ mice at two ages (young and adult) in both sexes, and to interrogate the role of AhR in colonic motility by integrating functional, histological, and molecular approaches.

## Materials and methods

### Experimental animals

The animal study was approved by the University of Valencia Ethics Committee for Research and Animal Welfare (license reference: A20231114124458, 2024-VSC-PEA-0010). Animals were housed at the Animal House Core Facility (University of Valencia) under controlled temperature with a relative humidity of 60% and artificial light-darkness cycles of 12 h.

Male and female C57BL/6J, wild type (WT) and AhR^−/−^ (Jackson Laboratory, Bar Harbor, ME, No. 002727;) were used in this study. Animals were housed in groups of 2–5 per cage and separated according to sex, age and genotype.

Mice were studied at two ages: young (4–6 months) and adult (12–13 months). Colon and blood samples were collected immediately after euthanasia by exsanguination for subsequent experimental analyses. For histological evaluation, colon segments were fixed in 4% paraformaldehyde, embedded in paraffin, sectioned, and processed for staining. Additional segments were snap-frozen in liquid nitrogen and stored at − 80 °C until protein and gene expression analyses. For lifespan assessment, an additional cohort was followed until natural death with systematic recording of causes of death. However, these data may underestimate specific causes of death due to limitations in post-mortem assessment. Body weight was monitored monthly.

The number of animals included in each experimental procedure is indicated in the corresponding figure legends and tables.

### Food and water intake

The mice had ad libitum access to food and water. Daily consumptions were quantified by weighing chow and water bottles every 24 h. Food intake was determined by calculating the daily difference in pellet weight, while water intake was assessed by weighing drinking bottles on consecutive days.

Mice were housed in groups of 2–5 per cage. The measured values were normalized by dividing by the number of mice housed in each cage.

### Body composition using DXA

Body composition analysis in mice was performed by dual-energy X-ray absorptiometry (DXA) using the InAlyzer system (Medikors Inc., Seongnam-si, Republic of Korea) at the Central Unit for Research in Medicine (UCIM), Faculty of Medicine, University of Valencia. The evaluation was conducted at two time points: 4–6 months of age (young group) and 12–13 months of age (adult group), approximately 2–3 days before euthanasia. The following skeletal and body parameters were assessed: bone mineral content (BMC) (g), bone mineral density (BMD) (g/cm^2^), bone mineral area (BMA) (cm^2^), bone mineral volume (BMV) (cm^3^), fat mass (g), lean mass (g), and fat and lean tissue composition (%).

During the procedure, animals were anesthetized with vaporized isoflurane (between 1 and 2%) and immobilized by fixing the limbs and tail with adhesive tape to ensure optimal image quality.

### Hematology

Following deep anesthesia with isoflurane, mice were euthanized by exsanguination via the inferior vena cava, and blood was collected into EDTA tubes for hematological analysis. Hematological parameters from young and adult wild-type (WT) and AhR^−/−^ mice were analyzed using a veterinary hematology analyzer (Hematology Element HT5; Scil Animal Care Company, Heska Group, Viernheim, Germany) located in Central Unit for Research in Medicine (UCIM), Faculty of Medicine, University of Valencia.

### Measurement of fecal parameters

For fecal collection, mice were housed in groups of 2–5 animals per cage. Every day at 12 p.m., 24-hour feces were collected in tubes, counted, and weighed using a precision balance (Mark330, BEL Engineering s.r.l., Monza MB, Italia). Subsequently, samples were dried at 100 °C for 30 min and reweighed. The number and weight of fecal pellets, measured before and after drying, were normalized to the number of mice per cage.

Fecal water content was calculated as:

$$\:Fecal\:water\:content\:\left(\%\right)\:=\:[\left(wet\:weight\hspace{0.17em}-\hspace{0.17em}dry\:weight\right)\:/\:wet\:weight]\:\:\times\:\:100$$

The Bristol Stool Scale was adapted to the mice model to study the length and consistency of the stools. For this purpose, the stool length was measured with a ruler, and fecal appearance was documented taking images under standardized lighting on a uniform white background.

Images were captured using a fixed-mounted digital camera under constant illumination conditions. The images were subsequently converted to 8-bit grayscale (0–255) and analyzed with a custom script developed in Python with OpenCV (OpenCV v.4.13.0.90). Segmentation was performed by Otsu thresholding to generate binary masks, isolate fecal contours, and extract grayscale intensity metrics (mean, standard deviation, and intensity distributions). Stool color is reported as arbitrary grayscale intensity units, where lower values indicate darker stools and higher values indicate lighter stools.

### Organ bath experiments

Following sacrifice, the colon was dissected from the abdominal cavity and placed on a petri dish containing physiological saline solution (0.9% NaCl). The fat and mesentery were carefully removed, and 1-cm-long segments of the proximal colon were obtained by cutting just below the cecum. Fecal contents were eliminated by gentle flushing with saline solution using a pipette. Fresh colon segments were mounted longitudinally between two metal hooks, attached at the oral and caudal ends connected to force transducers in an organ bath [20]. The bath contained 4 mL of physiological Krebs solution composed of (in mM): NaCl 115; KCl 4.6; MgCl_2_· 6H_2_O 1.2; CaCl_2_ 2.5; NaHCO_3_ 25; glucose 11.1 and EDTA disodium 0.01 and was continuously perfused with a gaseous solution of 95% O_2_ and 5% CO_2_ providing a pH between 7.3 and 7.4. The temperature was maintained up to 37 °C throughout the experiment. An initial tension of 0.5 g was set and allowed to equilibrate for 30 min before drugs were added. Changes in isometric tension were recorded with a PowerLab 8/30 data acquisition system (AD Instruments) using LabChart 7 software.

Spontaneous peristalsis was recorded during the final 15 min of the equilibration period. To evaluate the role of nitric oxide (NO) as an inhibitory neurotransmitter released by enteric neurons, the nitric oxide synthase (NOS) inhibitor *N*ω-nitro-L-arginine methyl ester (L-NAME, 10^− 4^ M) was added to the organ bath, and spontaneous peristalsis was measured after 15 min of incubation. In a separate set of experiments, cumulative concentrations of acetylcholine (ACh, 10^− 8^–10^− 4^ M) were administered to assess contractile responses, followed by cumulative concentrations of norepinephrine (NE, 10^− 9^–10^− 5^ M) to evaluate relaxation responses.

Spontaneous peristaltic activity was quantified as raw tension (mg) for amplitude and as frequency (Hz). ACh-induced responses were reported as raw tension values (mg), given that all tissue segments were prepared with identical dimensions and collected from the same anatomical region. Norepinephrine-induced relaxation was expressed as the percentage of relaxation relative to the ACh-induced contractile response.

The drugs used: ACh (Ref: A6625), NE (Ref: A9512), and L-NAME (Ref: N-575) were distributed by Sigma-Aldrich (St. Louis, MO, USA). Stock solutions were prepared in milliQ water and working dilutions were made in 0.9% NaCl.

### Histological study of colon

Fresh colon segments were collected and fixed in neutral-buffered formalin for 24 h. Subsequently, the samples were embedded in paraffin following a standard dehydration, clearing, and paraffin infiltration protocol using Leica ASP300 tissue processor (Leica Microsystems S.L.U., Barcelona, Spain). The resulting tissue sections were stained with hematoxylin and eosin (Agilent Technologies, Madrid, Spain). Microphotographs of the tissue were acquired using DMD108 photomicroscope (Leica Microsystems). Colon wall thickness was analyzed using FIJI software (ImageJ 2.0). Muscle layers were approximated as concentric circular structures, and mean thickness was calculated as the difference between radii derived from the areas enclosed by their inner and outer boundaries, using a modified version of a previously described method [21].

### Protein expression analysis

Colon segments were homogenized under cold conditions using an Ultra-Turrax homogenizer in a lysis buffer containing 66mM Tris - HCl, pH 7.5, 2% SDS, 5mM EDTA and 1% (v/v) protease/phosphatase inhibitors. The resulting homogenate was precipitated overnight at -20 °C and subsequently centrifuged at 14,000 rpm for 35 min at 4 °C. Additional centrifugation steps (20 min at 14,000 rpm and 4 °C) were repeated as necessary to carefully recover the supernatant while avoiding contamination from the upper lipid layer and the pellet. The protein concentration was determined using the bicinconic acid protein assay, BCA (Thermo Fisher, USA). The proteins (100 µg) were separated by denaturing sodium dodecyl sulfate–polyacrylamide gel electrophoresis (SDS-PAGE). For this purpose, the samples were loaded on Mini-PROTEAN TGX Stain-Free Precast Gels (Bio-Rad, USA) prepared with 7.5% polyacrylamide. A protein standard (Precision Plus Protein Dual Colour, Bio-Rad, USA) was used for molecular weight estimation. Electrophoresis was performed using a Mini-PROTEAN Tetra Cell system (Bio-Rad, USA) at a constant voltage of 140 V for 60 min. Proteins were subsequently transferred to polyvinylidene fluoride (PVDF) membranes previously activated with methanol. Membranes were then blocked for 1 h at room temperature in blocking buffer (5% skimmed milk, 0.1% Tween 20 in Tris-buffered solution), followed by overnight incubation at 4 °C with primary antibody against PGP9.5 (1:200, cat #ab8189, Abcam, UK) and neuronal nitric oxide synthase (nNOS/NOS1, 1:250, # ab76067 Abcam, UK). After incubation with the corresponding secondary antibodies: IRDye Donkey anti-rabbit 680RD (1:10 000, cat #925-680731, LI-COR) and Donkey anti-mouse 800WC (1:10 000, cat #925-32212, LI-COR) for 1 h at room temperature, the bands were detected using the fluorescence method and visualized with the Amersham ImageQuant TM 800 (Cytiva, Marlborough, MA, USA). Protein expression was quantified densitometrically using FIJI software (ImageJ 2.0) and normalized against total protein content using Stain-Free technology, based on polyacrylamide gels incorporating trihalo compounds that induce protein fluorescence during electrophoresis. This approach enables direct visualization of total protein in both the gel and membrane and provides a broader dynamic range with lower variability compared to conventional housekeeping protein normalization methods [22].

### Gene expression studies

The total RNA from colon tissue was extracted using 300 µL of TRIzol reagent. The RNA concentration was performed by Nanodrop 2000 (Agilent, Santa Clara, CA, USA), and the purity was evaluated with the 260/280 ratio. Reverse transcription quantitative polymerase chain reaction (RT-PCR) was performed for each of the following genes, using ready-to-use primer and probe sets pre-developed by Applied Biosystems (Foster City, CA, USA, TaqMan Gene Expression Assays) using Quantum Studio v5 (QuantStudioTM Design & Analysis Software v1.4.2), establishing the proper conditions. The housekeeping gene was *Gapdh* (Mm99999915_g1, Life Technologies, ThermoFisher Scientific), and the genes studied were *Nos1*, *Nos2*, *Aqp1*, *Aqp3*, *Aqp4*, *Aqp8*, *Il6* and *Il10* (reference numbers: Mm01208059_m1, Mm00440502_m1, Mm01326466_m1, Mm01208559_m1, Mm00802131_m1, Mm00431846_m1, Mm00446190_m1 and Mm01288386_m1 correspondingly Life Technologies, ThermoFisher Scientific). Relative gene expression was calculated using the 2^−ΔΔCt^ method [23].

### Statistical analysis

Data are presented as mean ± SD or mean ± SEM, as indicated in each figure legend.

The sample size (n) represents the number of animals included in each experimental group. Data normality was determined using the Shapiro-Wilk test. Multiple group comparisons were analyzed using two-way analysis of variance (ANOVA) followed by Bonferroni post hoc test. When data did not follow a normal distribution, the corresponding nonparametric tests were used.

Concentration–response curves were analyzed using two-way ANOVA, with genotype and concentration as factors, followed by a Bonferroni’s post hoc test. Survival analysis was conducted using the Kaplan–Meier method, and differences between survival curves were assessed using the Gehan–Breslow–Wilcoxon test. Statistical analyses and graphical representations were performed using GraphPad Prism version 10.3.1. Differences were considered statistically significant at *p* < 0.05.

## Results

### Survival curves

**Fig. 1:**
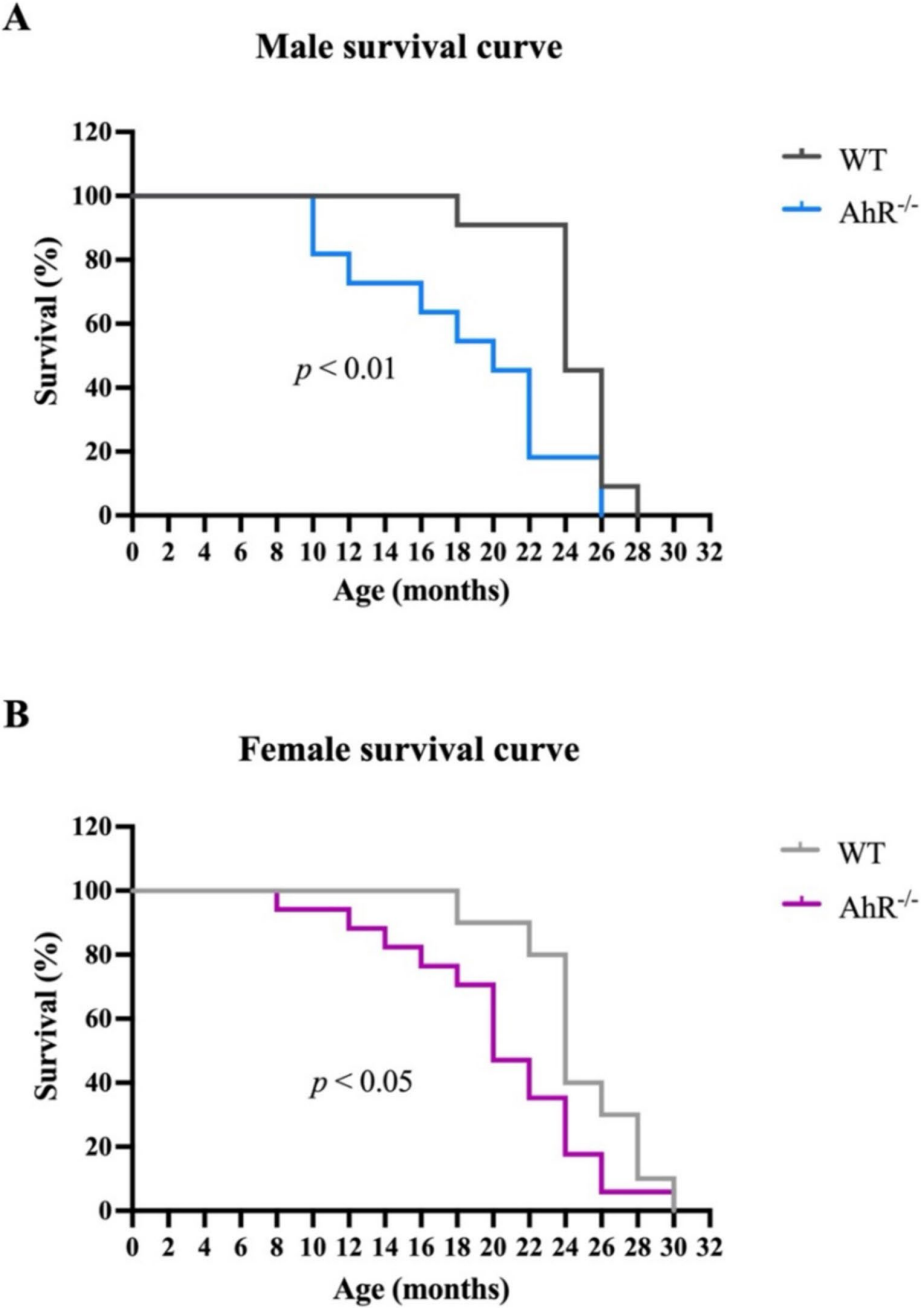
Survival curves of WT and AhR^−/−^ male (**A**) and female (**B**) mice. Statistical comparisons between AhR^−/−^ and WT groups were performed using the Gehan–Breslow–Wilcoxon test, and the corresponding *p* values are indicated in the figure. Sample size ranged from *n* = 10–17 per group

To determine whether AhR influences longevity, AhR^−/−^ mice and WT littermates were followed throughout life. AhR deficiency reduced survival in both sexes (Fig. 1A, B). Mortality in AhR^−/−^ males began at approximately 8 months, whereas WT males started to decline at ~ 16 months (Fig. 1A). Similarly, AhR^−/−^ females showed earlier decline beginning around 6 months compared with WT females (~ 16 months) (Fig. 1B).

In addition, the following parameters were calculated: the half-life of the mice (defined as the month in which survival is 50%) and maximum lifespan (month in which mortality is 100%). The results showed that the half-life of AhR^−/−^ animals was lower than WT animals in both males and females. In both sexes, the half-life in WT mice was 24 months (ratio 1.20) and in AhR^−/−^ group was 20 months (ratio 0.83) (Table 1). The maximal lifespan was 28 months in WT males and 26 months in AhR^−/−^ males. In females, this value was 30 months in both WT and AhR^−/−^ groups.

**Table 1:** Effect of receptor deficiency in lifespan metrics

	WT males	AhR^−/−^ males	WT females	AhR^−/−^ females
Half-life (ratio)	1.20 [0.520–2.768]	0.83 [ 0.362–1.922]	1.20 [0.549–2.621]	0.83 [0.382–1.820]
Maximal lifespan	28.00	26.00	30.00	30.00

Half-life: month at which the 50% of population has died. Maximal lifespan: month in which mortality is 100%

Half-life results shown with the interval confidence of ratio calculated at 95%

Causes of death differed between genotypes (Table 2). In WT males, splenic alterations were the most frequently recorded condition. In AhR^−/−^ males, rectal prolapse was prominent, alongside splenomegaly and blindness. In females, WT mice most frequently showed dermatological lesions, whereas AhR^−/−^ females presented rectal prolapse and blindness among recorded findings.

**Table 2:** Diseases and causes of death in WT and AhR^−/−^ males and females

	Males		Females	
	WT	AhR^−/−^	WT	AhR^−/−^
Cachexia	0/13 (0%)	0/28 (0%)	1/12 (8%)	1/31 (3%)
Ataxia	0/13 (0%)	0/28 (0%)	0/12 (0%)	2/31 (6%)
Splenomegaly or splenic tumor	1/13 (8%)	**3/28** **(11%)**	0/12 (0%)	0/31 (0%)
Nephromegaly	1/13 (8%)	0/28 (0%)	0/12 (0%)	1/31 (3%)
Bladder stones	0/13 (0%)	1/28 (4%)	0/12 (0%)	0/31 (0%)
Liver tumor or liver problems	0/13 (0%)	2/28 (7%)	0/12 (0%)	1/31 (3%)
Pancreatic tumor	0/13 (0%)	1/28 (4%)	0/12 (0%)	0/31 (0%)
Atopic dermatitis or skin ulcers	0/13 (0%)	1/28 (4%)	**2/12** **(17%)**	2/31 (6%)
Rectal prolapse	0/13 (0%)	**6/28** **(21%)**	0/12 (0%)	**4/31** **(13%)**
Blindness	0/13 (0%)	**3/28** **(11%)**	0/12 (0%)	**4/31** **(13%)**
Sudden death or unknown cause	11/13 (85%)	11/28 (39%)	9/12 (75%)	16/31 (52%)

### Changes in body weight across the lifespan

**Fig. 2:**
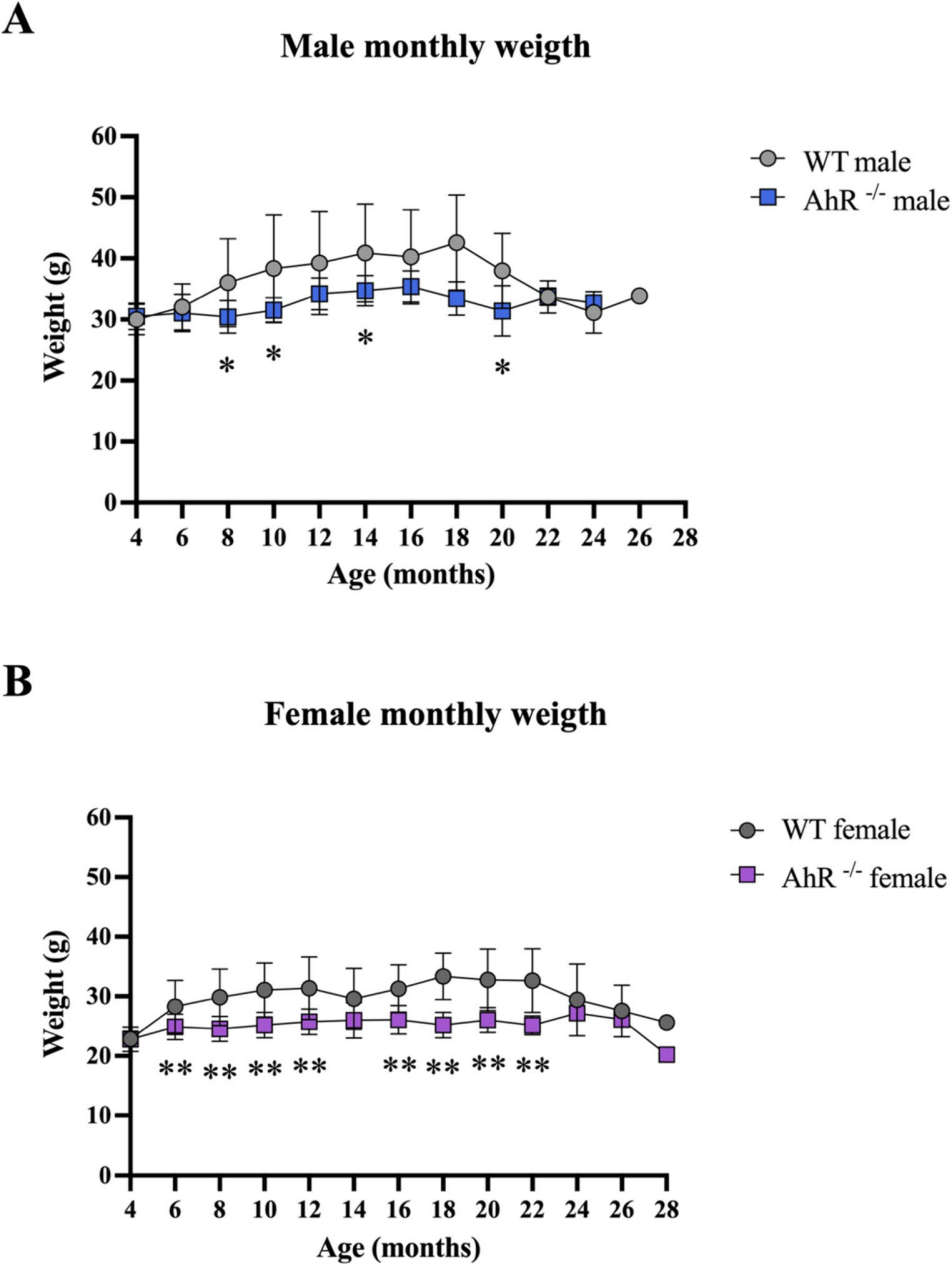
Body weight evolution. The figure shows the body weight (g) in WT and AhR^-/-^ males (**A**) and females (**B**) over a 28-month period. Data are expressed as mean ± SD and were analyzed using multiple t-test at each point of the curve (*n* = 10– 17 per group). *p < 0.05 and **p < 0.01 vs. the same-sex WT group

**Fig. 3:**
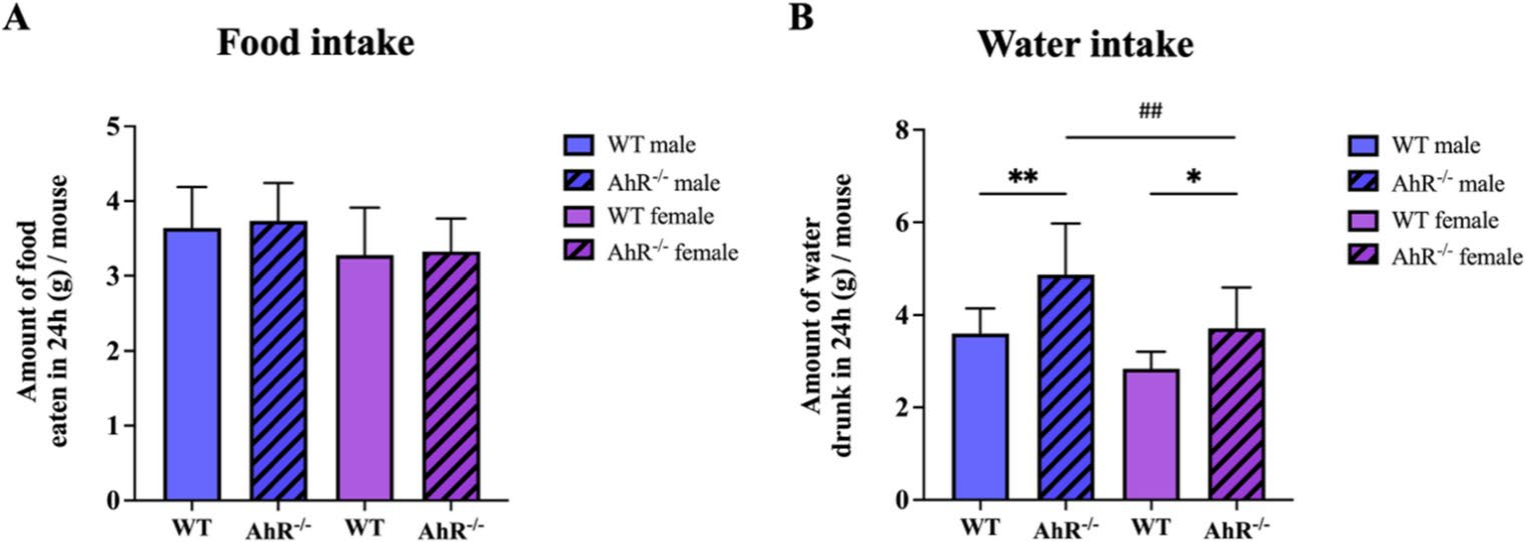
Food and water intake per mouse. The figure shows the food intake (**A**) and water intake (**B**) in WT and AhR^-/-^ male and female mice over a 24 -hour period. Data are expressed as mean ± SD and were analyzed using two-way ANOVA followed by a Bonferroni post hoc test (*n* = 12 per group; 4–5 mice per cage). *p < 0.05, **p < 0.01 vs. the same-sex WT group and ^##^*p* < 0.01 vs. AhR^-/-^ male

Body weight gain was reduced in AhR^−/−^ mice of both sexes, with a more pronounced effect in females (Fig. 2). Food consumption did not differ between genotypes in either sex (Fig. 3A). In contrast, AhR^−/−^ mice exhibited increased water intake in both sexes (Fig. 3B).

### Body composition and hematological profiles

DXA revealed no major changes in young AhR^−/−^ males. However, young AhR^−/−^ females showed reduced fat mass and fat percentage with a concomitant increase in lean percentage, together with lower bone parameters in comparison with WT female group (Table 3). In adults, AhR^−/−^ males displayed reduced fat mass and percentage and increased lean mass and percentage, while AhR^−/−^ females retained the reduced adiposity phenotype and showed reduced bone parameters (Table 4).

**Table 3:** Study of body composition in young male and female WT and AhR^−/−^ mice

	Young			
	Males		Females	
	WT	AhR^−/−^	WT	AhR^−/−^
Fat (g)	6.00 ± 2.28	4.91 ± 0.70	6.44 ± 2.91	3.74* ± 1.26
Lean (g)	23.10 ± 1.86	22.83 ± 0.39	18.64^##^ ± 1.11	17.93^##^ ± 1.66
Fat (%)	22.70 ± 9.05	18.46 ± 1.19	25.05 ± 9.21	14.87* ± 3.03
Lean (%)	79.26 ± 6.30	81.54 ± 1.19	73.66 ± 8.94	85.13* ± 3.03
BMC (g)	0.72 ± 0.08	0.67 ± 0.06	0.68 ± 0.03	0.62** ± 0.06
BMD (g/cm^2^)	0.07 ± 0.01	0.07 ± 0.01	0.07 ± 0.004	0.06 ± 0.004
BMA (cm^2^)	10.30 ± 0.37	9.97 ± 0.67	9.69^##^ ± 0.56	8.62*^#^ ± 0.58
BMV (cm^3^)	0.23 ± 0.03	0.22 ± 0.02	0.22 ± 0.01	0.19* ± 0.02

*BMC* Bone Mineral Content, *BMD* Bone Mineral Density, *BMA* Bone Mineral Area, *BMV* Bone Mineral Volume. Data are presented as mean ± SD and were analyzed using two-way ANOVA followed by a Bonferroni post hoc test or Dunn’s test for non-parametric data (*n* = 4–11 per group). *p < 0.05 and **p < 0.01 vs. the same-sex WT group; ^#^*p* < 0.05, ^##^*p* < 0.01 vs. male of the same genotype

**Table 4:** Study of body composition in adult male and female WT and AhR^−/−^ mice

	Adult			
	Males		Females	
	WT	AhR^−/−^	WT	AhR^−/−^
Fat (g)	13.93 ± 7.65	7.19* ± 1.67	14.84 ± 2.94	4.79** ± 0.67
Lean (g)	23.90 ± 1.55	25.90* ± 1.43	20.09 ± 2.51	19.52^#^ ± 0.63
Fat (%)	34.91 ± 13.05	21.66* ± 4.60	40.68^##^ ± 6.80	20.57** ± 1.58
Lean (%)	65.09 ± 13.05	78.34* ± 4.60	57.61 ± 6.50	79.44** ± 1.58
BMC (g)	0.71 ± 0.19	0.80 ± 0.13	0.77 ± 0.05	0.68* ± 0.07
BMD (g/cm^2^)	0.07 ± 0.01	0.08 ± 0.01	0.07 ± 0.01	0.06 ± 0.01
BMA (cm^2^)	10.99 ± 1.43	10.44 ± 1.02	11.91 ± 1.65	10.17* ± 0.43
BMV (cm^3^)	0.24 ± 0.06	0.25 ± 0.04	0.24 ± 0.02	0.21* ± 0.03

*BMC* Bone Mineral Content, *BMD* Bone Mineral Density, *BMA* Bone Mineral Area, *BMV* Bone Mineral Volume. Data are presented as mean ± SD and were analyzed using two-way ANOVA followed by a Bonferroni post hoc test or Dunn’s test for non-parametric data (*n* = 4–8 per group). *p < 0.05 and **p < 0.01 vs. the same-sex WT group; ^#^*p* < 0.05 and ^##^*p* < 0.01 vs. male of the same genotype

Hematological analyses indicated age-, sex- and genotype-dependent alterations in leukocyte and erythroid parameters. In young AhR^−/−^ males, neutrophil, monocyte, and eosinophil counts were reduced, accompanied by a lower percentage of neutrophils and eosinophils, an increased percentage of monocytes, and elevated erythroid indices, including MCH, RDW-CV, and trends toward increased HGB and RBC values (Table 5). Young AhR^−/−^ females exhibited increased WBC, lymphocyte, and eosinophil counts, together with a reduced neutrophil percentage and enhanced erythroid parameters, including HGB, HCT, MCV, MCH, and MCHC (Table 5). In adult AhR^−/−^ males, WBC, neutrophil, and monocyte counts were increased, accompanied by higher HGB and MCV values and a reduction in eosinophil percentage (Table 6). In contrast, adult AhR^−/−^ females showed decreased lymphocyte and monocyte counts, reduced lymphocyte percentages, lower basophil counts and percentages, reduced RBC levels, and increased neutrophil percentages and MCV values (Table 6).

**Table 5:** Hematological study in young male and female WT and AhR^−/−^ mice

	Young			
	Males		Females	
	**WT**	**AhR** ^**−/−**^	**WT**	**AhR** ^**−/−**^
WBC (10^9^/L)	5.23 ± 1.93	4.70 ± 2.02	4.15 ± 1.36	6.32* ± 2.32
Neutrophils (10^9^/L)	1.32 ± 0.82	0.59* ± 0.22	0.79 ± 0.43	0.90 ± 0.28
Lymphocytes (10^9^/L)	3.82 ± 1.48	4.16 ± 1.90	2.62 ± 1.49	5.16* ± 2.18
Monocytes (10^9^/L)	0.22 ± 0.10	0.12** ± 0.05	0.08^##^ ± 0.01	0.13 ± 0.05
Eosinophils (10^9^/L)	0.11 ± 0.07	0.04* ± 0.03	0.06 ± 0.02	0.13*^##^ ± 0.02
Basophils (10^9^/L)	0.007 ± 0.02	0.007 ± 0.008	0.005 ± 0.01	0.005 ± 0.01
Neutrophils (%)	21.01 ± 10.45	11.99* ± 4.06	28.63 ± 6.87	17.52* ± 6.42
Lymphocytes (%)	73.51 ± 12.25	80.76 ± 9.71	72.00 ± 14.81	78.49 ± 7.82
Monocytes (%)	2.88 ± 1.01	5.26* ± 0.48	2.22 ± 0.93	4.79* ± 1.38
Eosinophils (%)	2.51 ± 1.15	1.13* ± 0.25	2.14 ± 0.90	1.54 ± 0.32
Basophils (%)	0.21 ± 0.15	0.14 ± 0.13	0.05^#^ ± 0.05	0.1 ± 0.06
RBC (10^12^/L)	9.94 ± 0.52	10.29 ± 1.44	8.82 ± 1.54	9.89 ± 1.14
HGB (g/dL)	15.16 ± 1.09	17.38 ± 1.88	13.67 ± 2.32	16.76* ± 1.99
HCT (%)	44.16 ± 2.23	45.50 ± 3.31	41.39 ± 4.12	46.97** ± 2.94
MCV (fL)	42.67 ± 1.58	44.53 ± 2.05	44.79^#^ ± 0.46	45.86** ± 0.78
MCH (pg)	15.22 ± 0.36	16.41*** ± 0.32	15.52 ± 0.43	16.84*** ± 0.52
MCHC (g/dL)	35.77 ± 1.82	36.76 ± 1.09	34.69 ± 0.71	36.74* ± 0.98
RDW - CV (%)	14.26 ± 1.30	15.30* ± 0.46	13.42 ± 0.29	14.11^#^ ± 0.63
PLT (10^9^/L)	726.14 ± 334.44	779.50 ± 356.23	613.11 ± 437.05	594.29 ± 55.26
MPV (fL)	5.01 ± 0.42	5.07 ± 0.42	5.63^##^ ± 0.24	5.48 ± 0.17

*WBC* White Blood Cells, *RBC* Red Blood Cells, *HGB* Hemoglobin, *HCT* Hematocrit, *MCV* Medium Corpuscular Volume, *MCH* Mean Corpuscular Hemoglobin, *MCHC* Mean Corpuscular Hemoglobin Concentration, *RDW-CV* Red Cell Distribution Width, *PLT* Platelets, *MPV* Medium Platelet Volume. Data are presented as mean ± SD and were analyzed using two-way ANOVA followed by a Bonferroni post hoc test or Dunn’s test for non-parametric data (*n* = 5–10 per group). *p < 0.05, **p < 0.01 and ***p < 0.001 vs. the same-sex WT group; ^#^*p* < 0.05 and ^##^ *p* < 0.01 vs. male of the same genotype

**Table 6:** Hematological study in adult male and female WT and AhR^−/−^ mice

	Adult			
	Males		Females	
	**WT**	**AhR** ^**−/−**^	**WT**	**AhR** ^**−/−**^
WBC (10^9^/L)	4.81 ± 1.75	9.58* ± 3.68	6.79 ± 1.31	5.72^##^ ± 1.20
Neutrophils (10^9^/L)	0.96 ± 0.44	2.23* ± 0.97	1.56 ± 0.15	1.99* ± 0.31
Lymphocytes (10^9^/L)	3.29 ± 1.28	6.65 ± 3.14	4.86 ± 1.42	3.08^##^ ± 1.22
Monocytes (10^9^/L)	0.14 ± 0.04	0.44* ± 0.27	0.25 ± 0.06	0.13*^##^ ± 0.01
Eosinophils (10^9^/L)	0.04 ± 0.02	0.05 ± 0.02	0.08 ± 0.02	0.07 ± 0.07
Basophils (10^9^/L)	0.006 ± 0.005	0.02 ± 0.01	0.04^#^ ± 0.02	0.01* ± 0.01
Neutrophils (%)	17.24 ± 2.38	23.79 ± 10.48	26.88 ± 3.13	36.11^#^ ± 5.81
Lymphocytes (%)	74.30 ± 5.40	74.71 ± 6.48	69.56 ± 6.82	58.98*^##^ ± 6.02
Monocytes (%)	3.17 ± 0.64	3.88 ± 1.61	3.33 ± 0.38	3.39 ± 1.12
Eosinophils (%)	1.13 ± 0.76	0.64* ± 0.22	1.24 ± 0.36	1.36 ± 1.24
Basophils (%)	0.20 ± 0.06	0.20 ± 0.11	0.68^###^ ± 0.23	0.28*** ± 0.16
RBC (10^12^/L)	7.86 ± 1.43	8.21 ± 0.99	9.65^#^ ± 0.89	8.27* ± 0.69
HGB (g/dL)	11.15 ± 1.62	13.43** ± 1.85	15.93^###^ ± 0.64	13.89 ± 0.79
HCT (%)	35.00 ± 6.60	36.96 ± 6.16	43.90 ± 1.36	38.70 ± 3.12
MCV (fL)	44.50 ± 1.60	46.37* ± 1.23	44.18 ± 1.43	46.87** ± 1.64
MCH (pg)	15.28 ± 0.31	16.02 ± 0.78	15.92 ± 0.46	16.50 ± 0.38
MCHC (g/dL)	34.37 ± 0.99	34.56 ± 1.43	36.06 ± 0.62	35.27 ± 1.28
RDW - CV (%)	13.77 ± 0.86	15.14 ± 1.42	13.18 ± 0.36	13.97 ± 1.12
PLT (10^9^/L)	971.25 ± 88.65	935.78 ± 316.54	884.75 ± 230.04	838.80 ± 192.42
MPV (fL)	5.07 ± 0.31	5.30 ± 0.36	5.44 ± 0.53	5.43 ± 0.28

*WBC* White Blood Cells, *RBC* Red Blood Cells, *HGB* Haemoglobin, *HCT* Haematocrit, *MCV* Medium Corpuscular Volume, *MCH* Mean Corpuscular Haemoglobin, *MCHC* Mean Corpuscular Haemoglobin Concentration, *RDW-CV* Red Cell Distribution Width, *PLT* Platelets, *MPV* Medium Platelet Volume. Data are presented as mean ± SD and were analyzed using two-way ANOVA followed by a Bonferroni post hoc test or Dunn’s test for non-parametric data (*n* = 4–10 per group). *p < 0.05, **p < 0.01 ***p < 0.001 vs. the same-sex WT group; ^#^*p* < 0.05, ^##^ *p* < 0.01, ^##^ *p* < 0.001 vs. male of the same genotype

### Fecal output

**Fig. 4:**
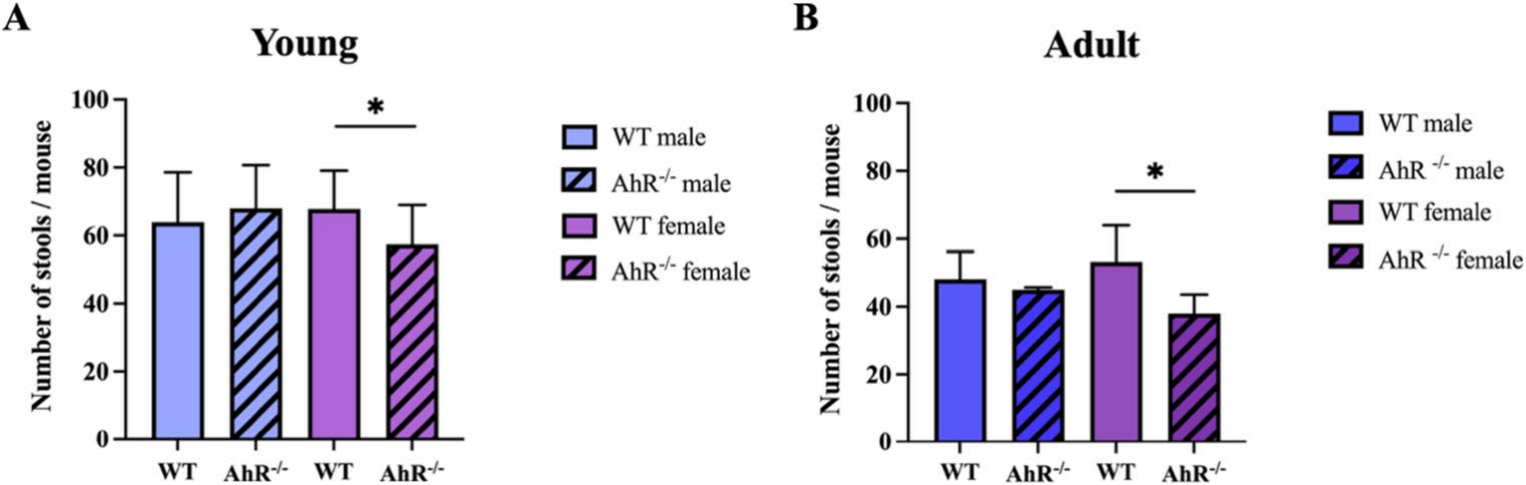
Total number of stools per mouse. The figure shows the total number of stools excreted by WT and AhR^−/−^ male and female mice in young (**A**) and adult (**B**) groups. Data are expressed as mean ± SD and were analyzed using two-way ANOVA followed by a Bonferroni post hoc test (*n* = 4 − 17 per group; 2–5 mice per cage). * *p* < 0.05 vs. WT female

Fecal output exhibited sex-specific effects. No differences in the number of stools were observed in males, either in young individuals or in adults (Fig. 4A and B). In contrast, AhR^−/−^ females showed a reduced number of stools in both young and adult groups (Fig. 4A and B).

### Bristol stool scale

**Fig. 5:**
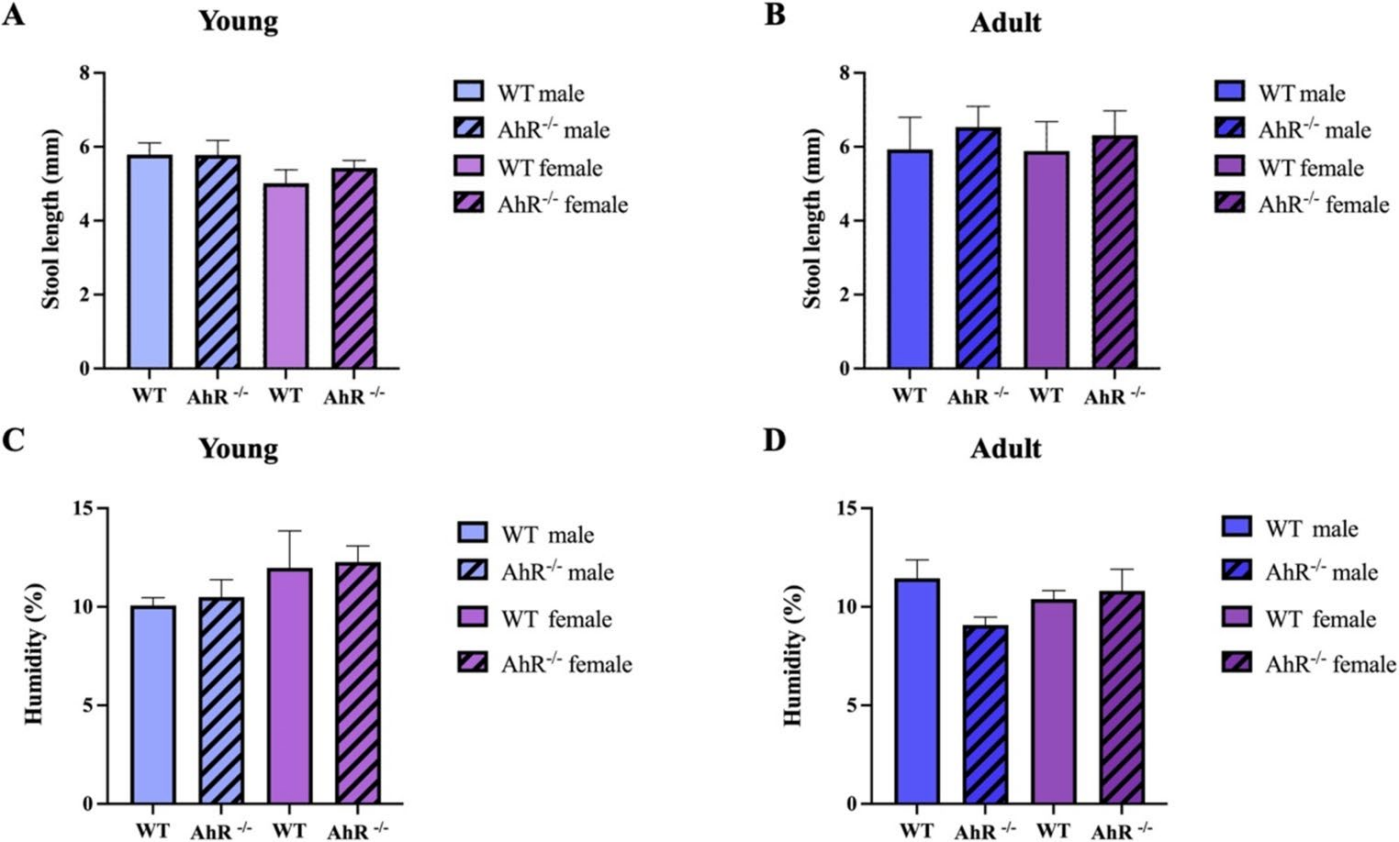
Bristol Stool Scale (I): stool length and moisture. The figure shows stool length (mm) of 24-hour fecal output in WT and AhR^−/−^ male and female mice in young (**A**) and adult (**B**) groups. Fecal humidity (%) measured over 24 h in WT and AhR^−/−^ male and female in young (**C**) and adult (**D**) groups. Data are expressed as mean ± SD (*n* = 4–17 per group; 2–5 mice per cage)

Fecal length and moisture content did not differ across genotypes, sexes or ages (Fig. 5A-D).

**Fig. 6:**
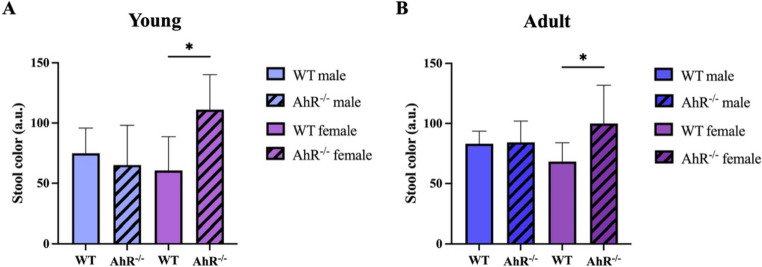
Bristol Stool Scale (II): stool color. The figure shows stool color (a.u.) of 24-hour fecal output in WT and AhR^-/-^ male and female mice in young (**A**) and adult (**B**) groups. Data are expressed as mean ± SD and were analyzed using two-way ANOVA followed by a Bonferroni post hoc test (*n* = 4–17 per group; 2–5 mice per cage). *p < 0.05 vs. WT female

Stool color, however, revealed sex-specific effects. In males the grayscale-based color did not differ between WT and AhR^−/−^ at either age (Fig. 6A, B). In contrast, AhR^−/−^ females showed increased grayscale intensity values (lighter stools) at both ages (Fig. 6A, B). No differences in stool hardness were observed, although this parameter was assessed observationally (see Supplementary material, Figure S1 and S2).

### Spontaneous colonic peristalsis

**Fig. 7:**
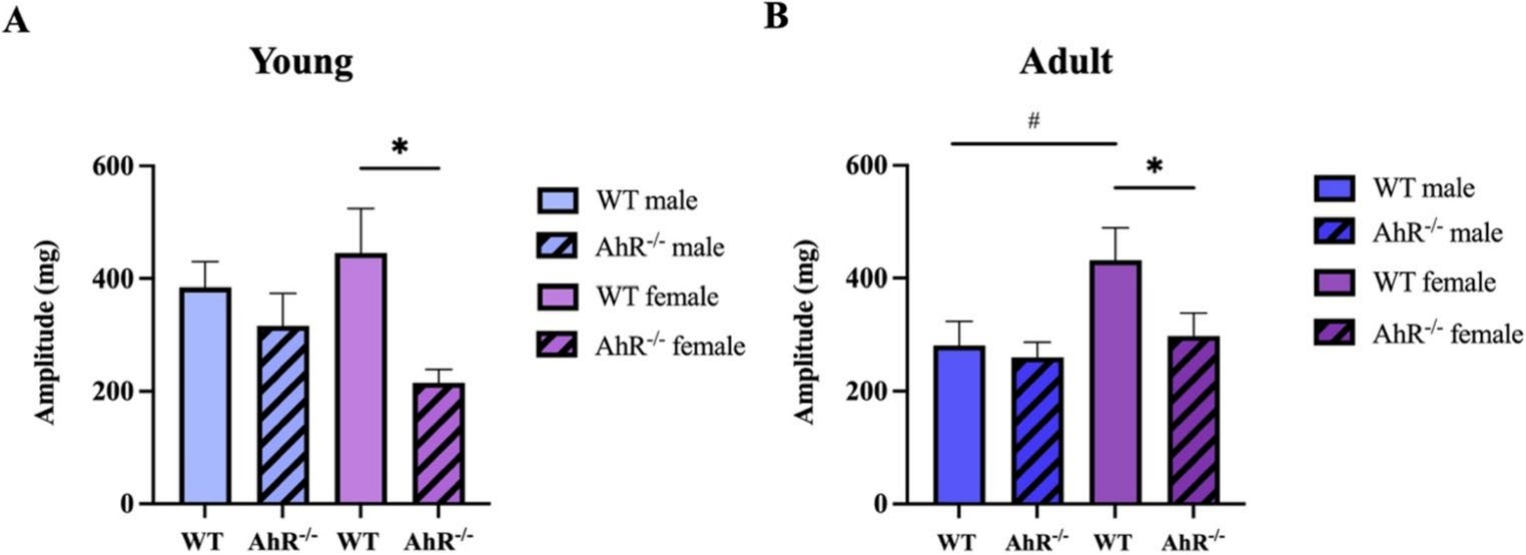
Spontaneous peristalsis. Amplitude (mg of tension) was measured in 1-cm-long colon segments of WT and AhR^−/−^ male and female mice in young (**A**) and adult (**B**) groups. Data are expressed as mean ± SEM and were analyzed using two-way ANOVA followed by a Bonferroni post hoc test (*n* = 5–15 per group). *p < 0.05 vs. WT female; ^#^*p* < 0.05 vs. WT male

Spontaneous peristalsis recorded ex vivo showed no differences between WT and AhR^−/−^ males at either age (Fig. 7A, B). In females, spontaneous peristaltic amplitude was reduced in AhR^−/−^ mice compared with WT in both young and adult groups (Fig. 7A, B).

AhR^−/−^ genotype did not change the frequency of spontaneous peristalsis (Fig. S3 Material Supplementary).

**Fig. 8:**
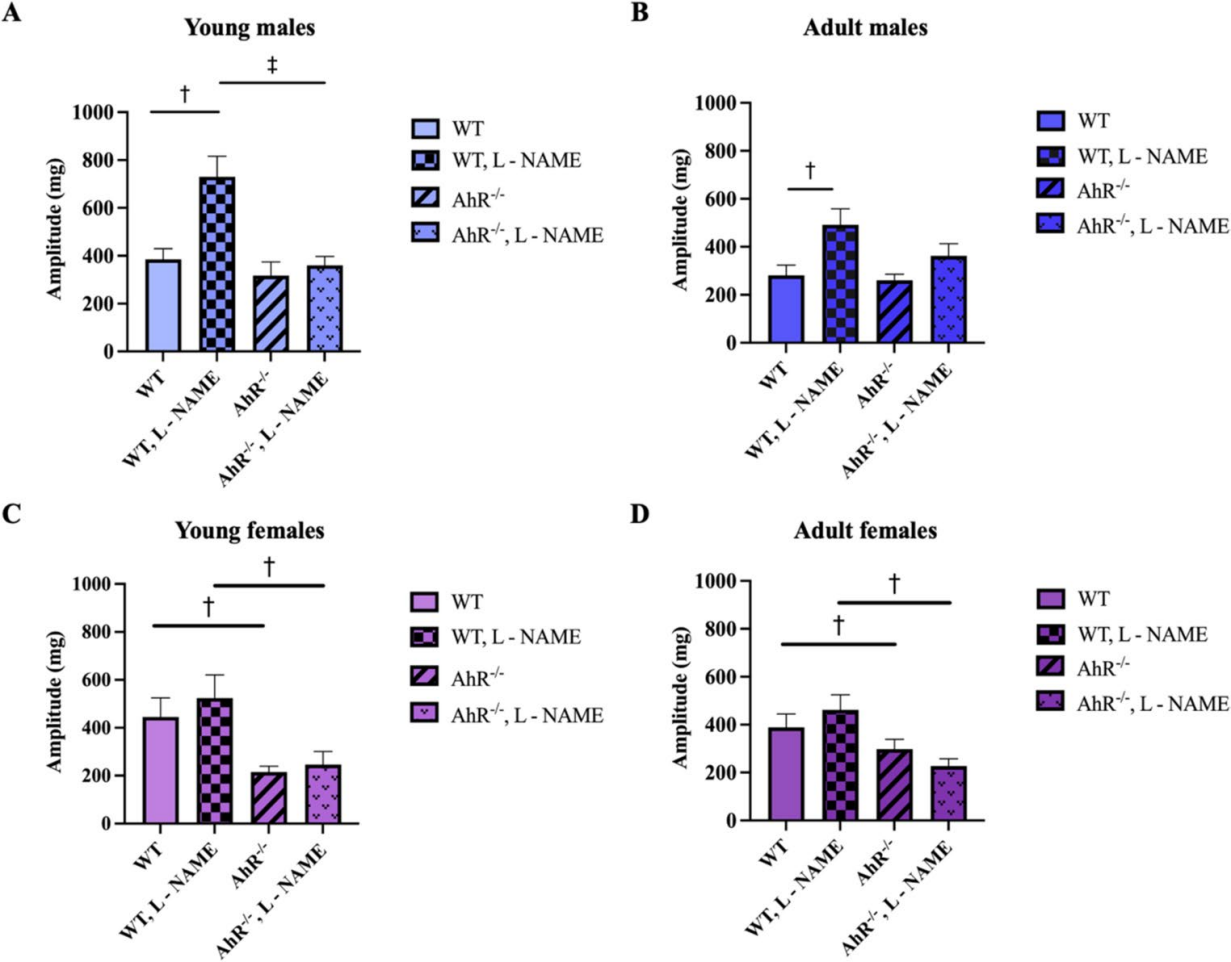
Spontaneous colon peristalsis after 15 min with or without incubation with L-NAME. The amplitude (mg tension) was measured in 1-cm-long colon segments of WT and AhR^−/−^ mice in young (**A** and **C**) and adult (**B** and **D**) groups. Data are expressed as mean ± SEM and were analyzed using two-way ANOVA followed by a Bonferroni post hoc test (*n* = 5–10 per group). † *p* < 0.05, ‡ *p* < 0.01

In WT males, L-NAME increased spontaneous peristaltic amplitude in both young and adult groups, supporting a tonic inhibitory role of NO in colonic motility. This response was absent in AhR^−/−^ males (Fig. 8A, B), suggesting impaired nitrergic modulation in the absence of AhR. In females, L-NAME did not significantly alter spontaneous peristalsis in either genotype or age group (Fig. 8C, D), in contrast to the effects observed in males.

### Colon contractile response

**Fig. 9:**
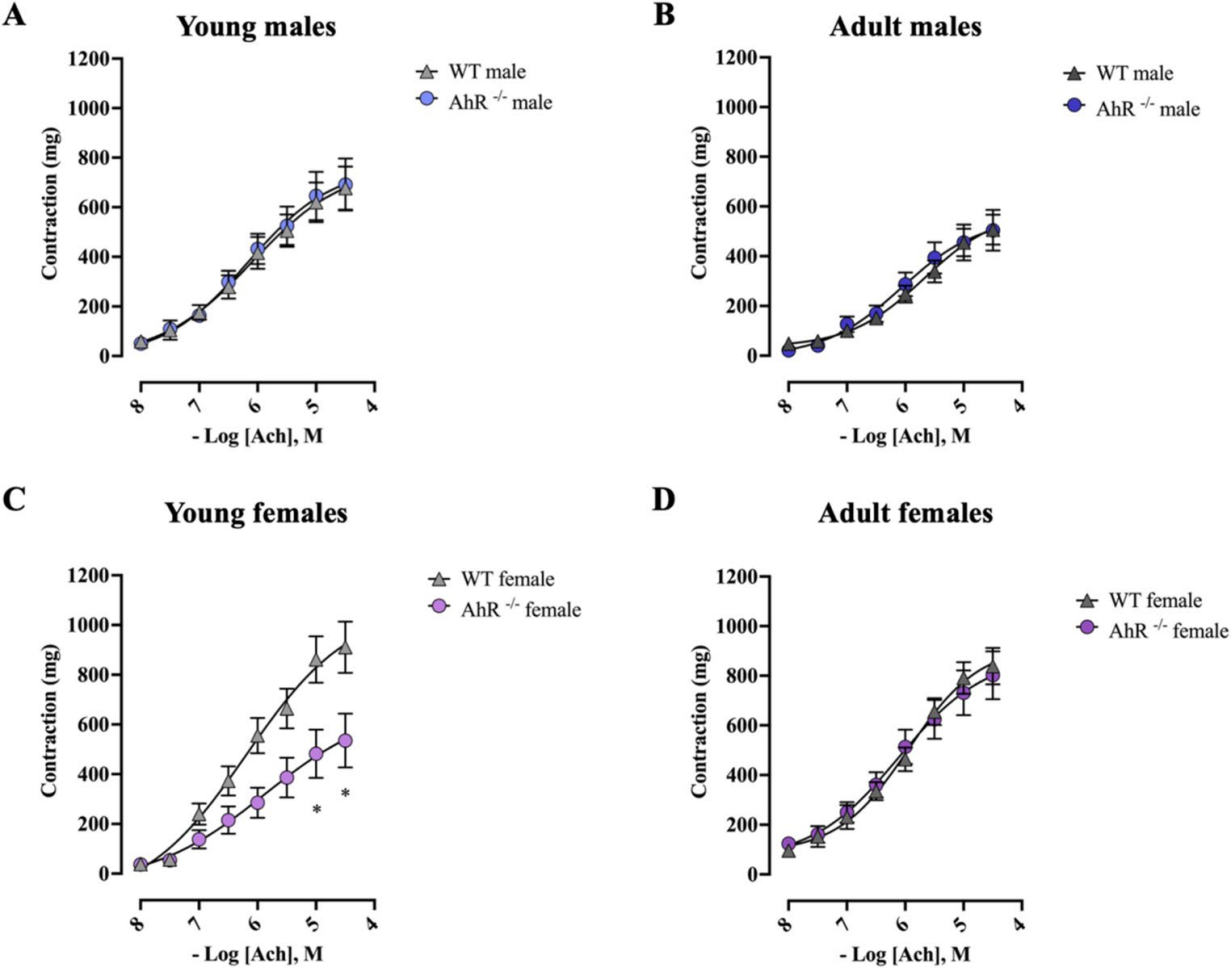
Concentration - response curves to acetylcholine in proximal colon. The data show the contractile responses (expressed as mg of tension) of 1-cm colon segments to acetylcholine (ACh, 10^− 8^ to 10^− 4^ M) in WT and AhR^−/−^ mice in young (**A** and **C**) and adult (**B** and **D**) groups. Data are expressed as mean ± SEM and were analyzed using two-way ANOVA followed by a Bonferroni post hoc test (*n* = 6–10 per group). *p < 0.05 vs. WT female

Muscarinic contraction assessed by ACh concentration-response curves showed no genotype differences in males at either age (Fig. 9A, B). In females, ACh-induced contraction was reduced in young AhR^−/−^ mice compared with WT (Fig. 9C), whereas no differences were observed in adults (Fig. 9D).

### Colon relaxation response

**Fig. 10:**
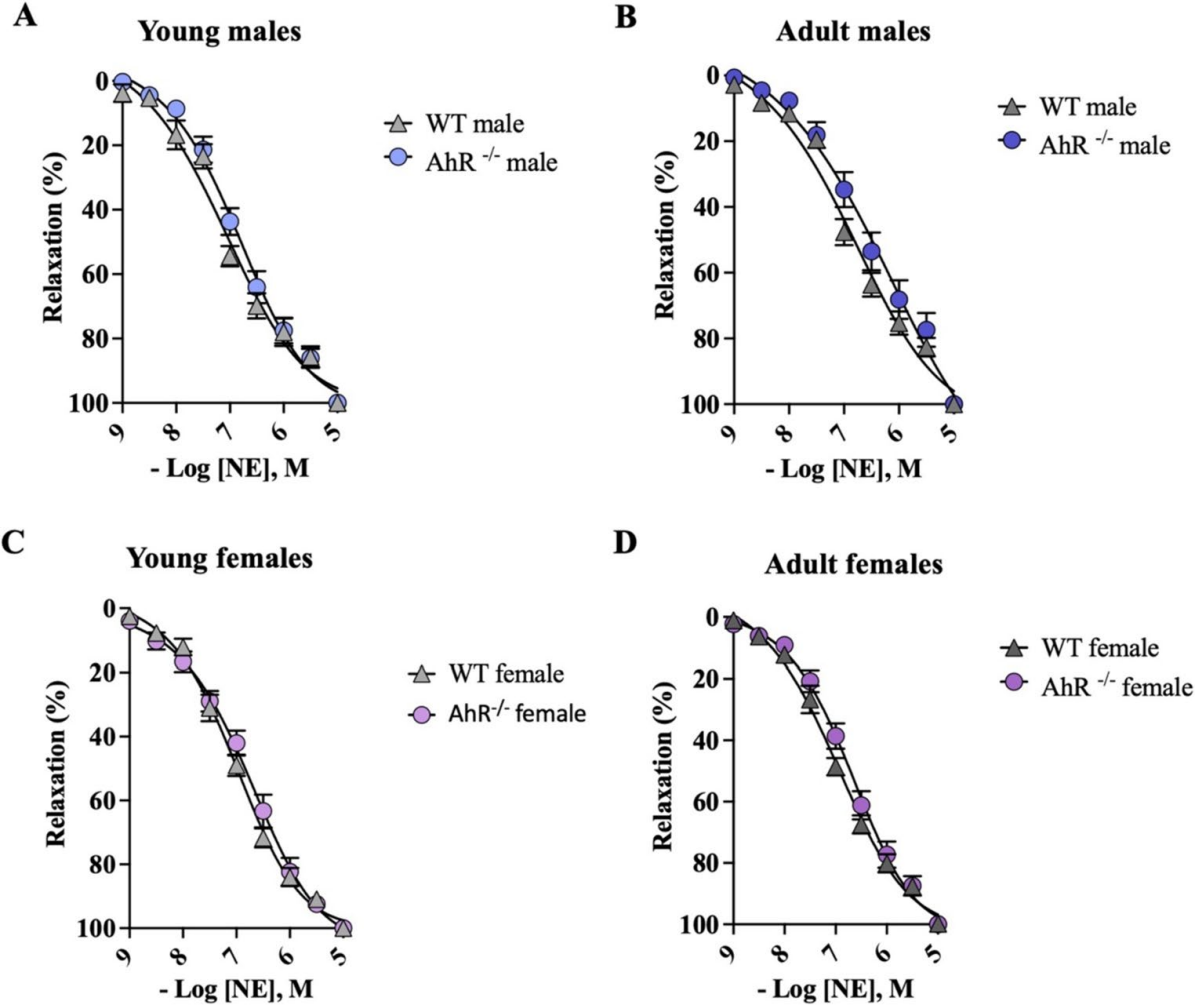
Concentration-response curves to norepinephrine in proximal colon. The data show the relaxing responses to norepinephrine (NE, 10^− 9^ to 10^− 5^ M) in WT and AhR^−/−^ mice in young (**A** and **C**) and adult (**B** and **D**) groups. Data are expressed as mean ± SEM and were analyzed using two-way ANOVA followed by a Bonferroni post hoc test (*n* = 6–8 per group)

Adrenergic relaxation to NE was not altered by AhR deficiency in any group (Fig. 10).

### Histological study in colon

**Fig. 11:**
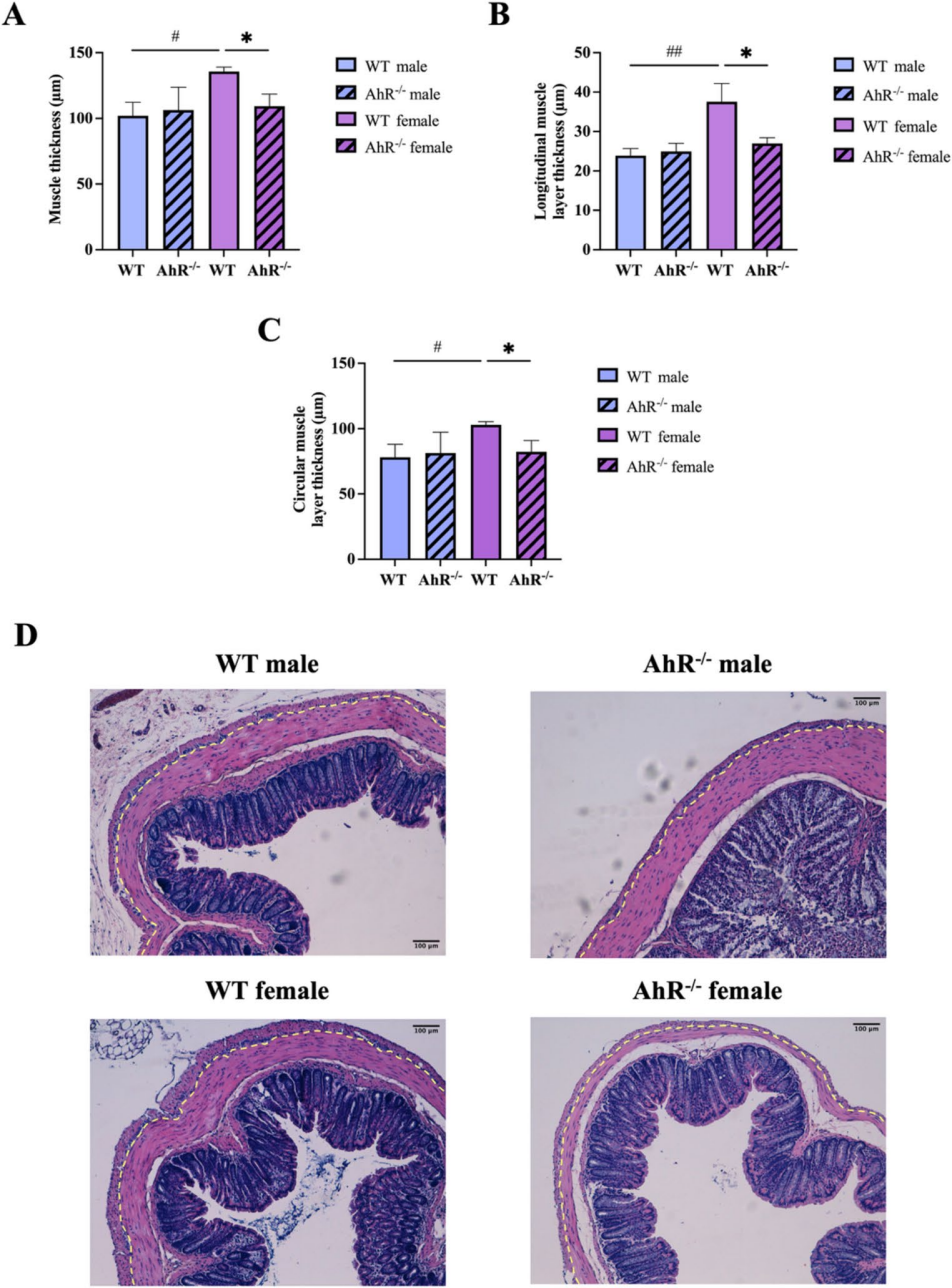
Histological study in colon segments of young male and female from WT and AhR^−/−^ mice. The figure shows, in young male and female mice, the total muscle thickness (**A**), the longitudinal muscle layer thickness (**B**) and the circular muscle layer thickness (**C**). In panel **D**, representative hematoxylin and eosin-stained images of each group are shown (scale bar = 100 μm). Data are expressed as mean ± SEM and were analyzed using two-way ANOVA test (*n* = 5–9 per group). *p < 0.05 vs. WT female, ^#^*p* < 0.05 and ^##^*p* < 0.01 vs. WT male

**Fig. 12:**
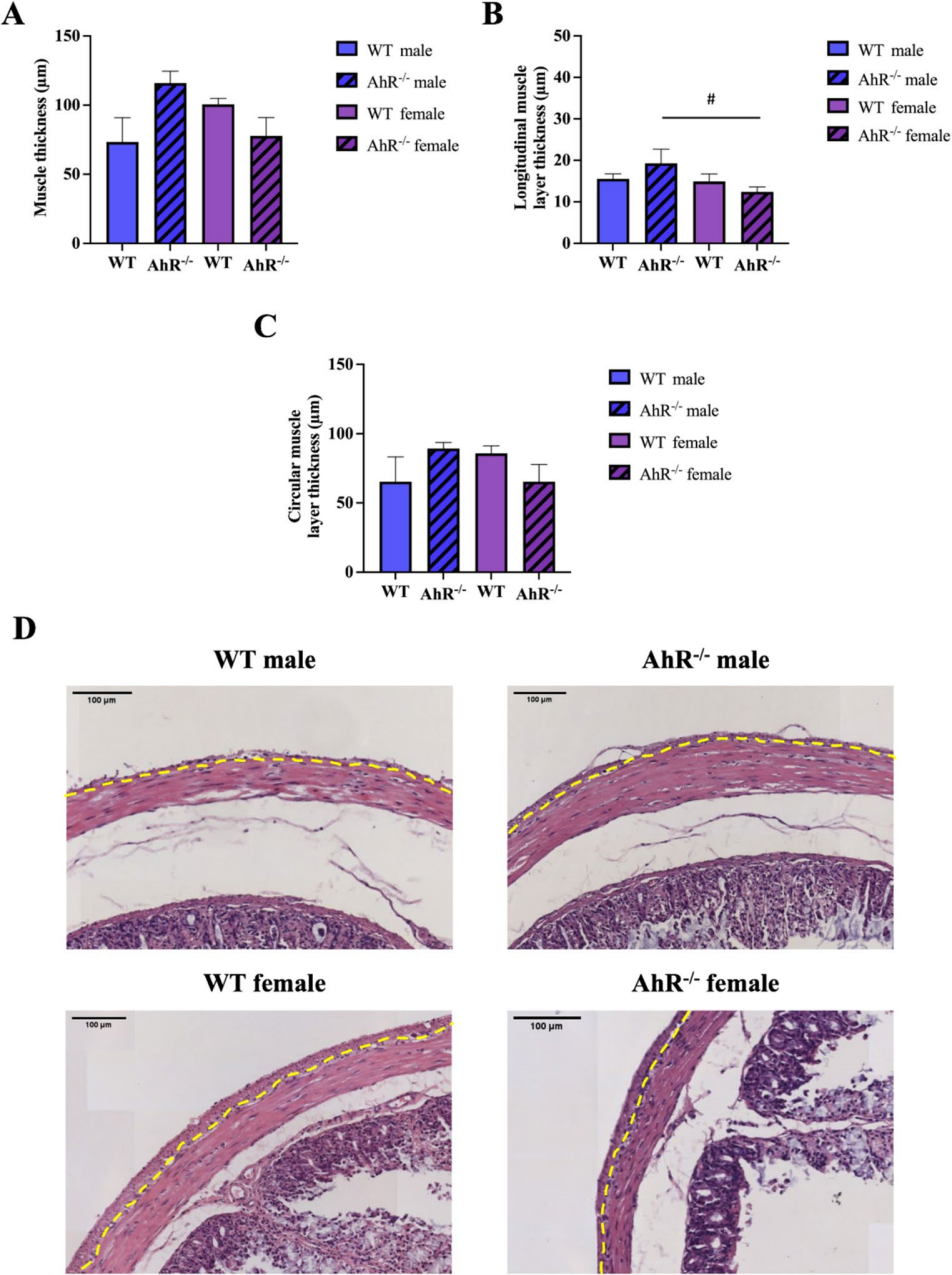
Histological study in colon segments of adult male and female from WT and AhR^−/−^ mice. The figure shows, in adult male and female mice, the total muscle thickness (**A**), the longitudinal muscle layer thickness (**B**) and the circular muscle layer thickness (**C**). In panel **D**, representative hematoxylin and eosin-stained images of each group are shown (scale bar = 100 μm). Data are expressed as mean ± SEM and data were analyzed using two-way ANOVA test (*n* = 5–12 per group). ^#^*p* < 0.05 vs. AhR^−/−^ male

Histological analysis revealed no differences in total, circular, or longitudinal muscle thickness in young (Fig. 11A–D) and adult (Fig. 12A–D) males. In young females, AhR^−/−^ mice displayed reduced total muscle thickness and reduced circular and longitudinal layers (Fig. 11A–D). No significant thickness changes were detected in adult females (Fig. 12A–D).

### Protein expression of enteric neuronal markers

Protein levels of PGP9.5, a general marker of enteric nerve fibers and synaptic terminals, and neuronal nitric oxide synthase (NOS1), a marker of nitrergic neurons, were analyzed in colonic tissue.

**Fig. 13:**
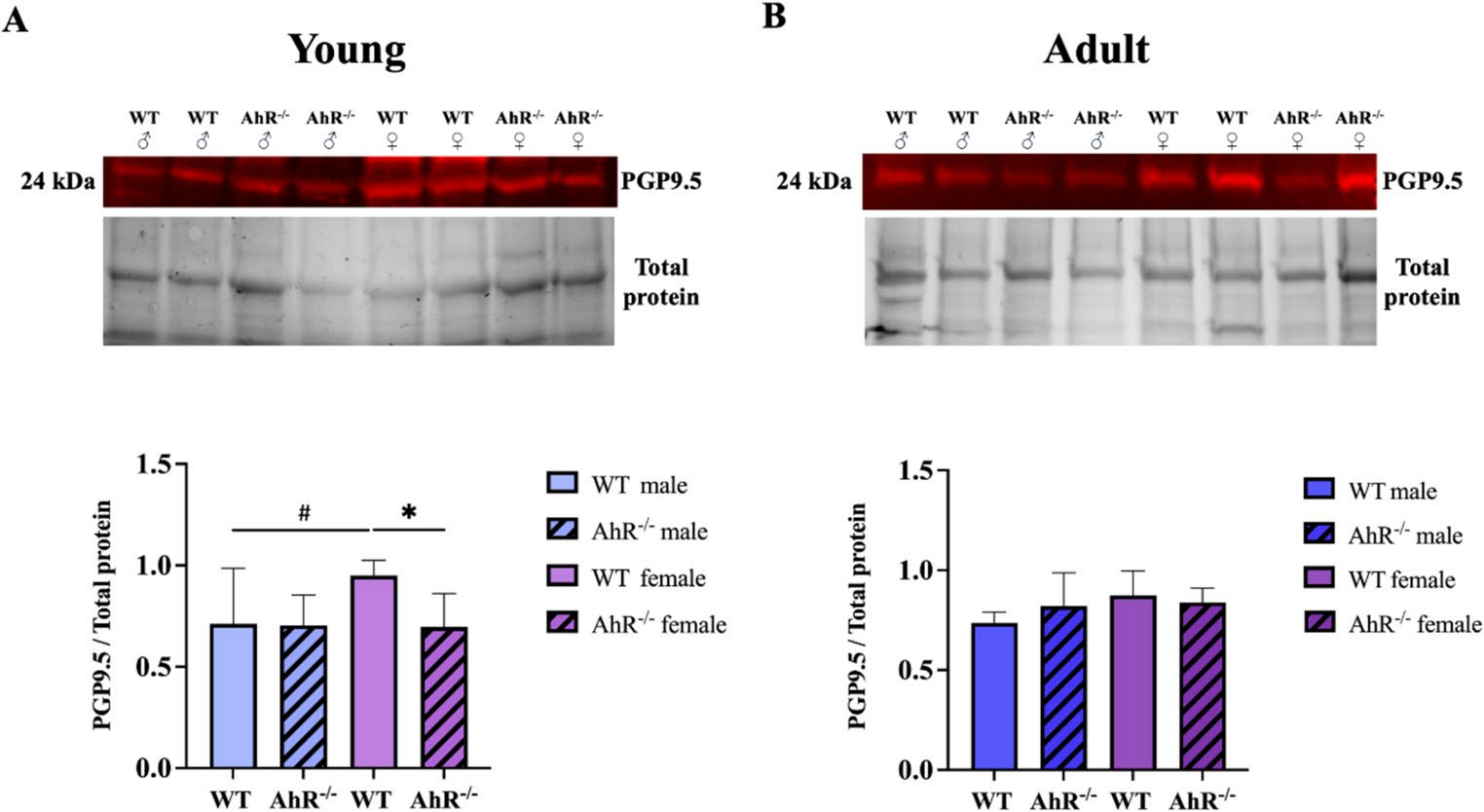
PGP9.5 protein expression in colon. The figure shows representative immunoblots and PGP9.5 protein expression in WT and AhR^−/−^ male and female mice in young (**A**) and adult (**B**) groups. Densitometric values were normalized to total protein using Stain-Free technology. Data are expressed as mean ± SD and were analyzed using two-way ANOVA followed by a Bonferroni post hoc test (*n* = 5–6 per group). *p < 0.05 vs. WT female, ^#^*p* < 0.05 vs. WT male

**Fig. 14:**
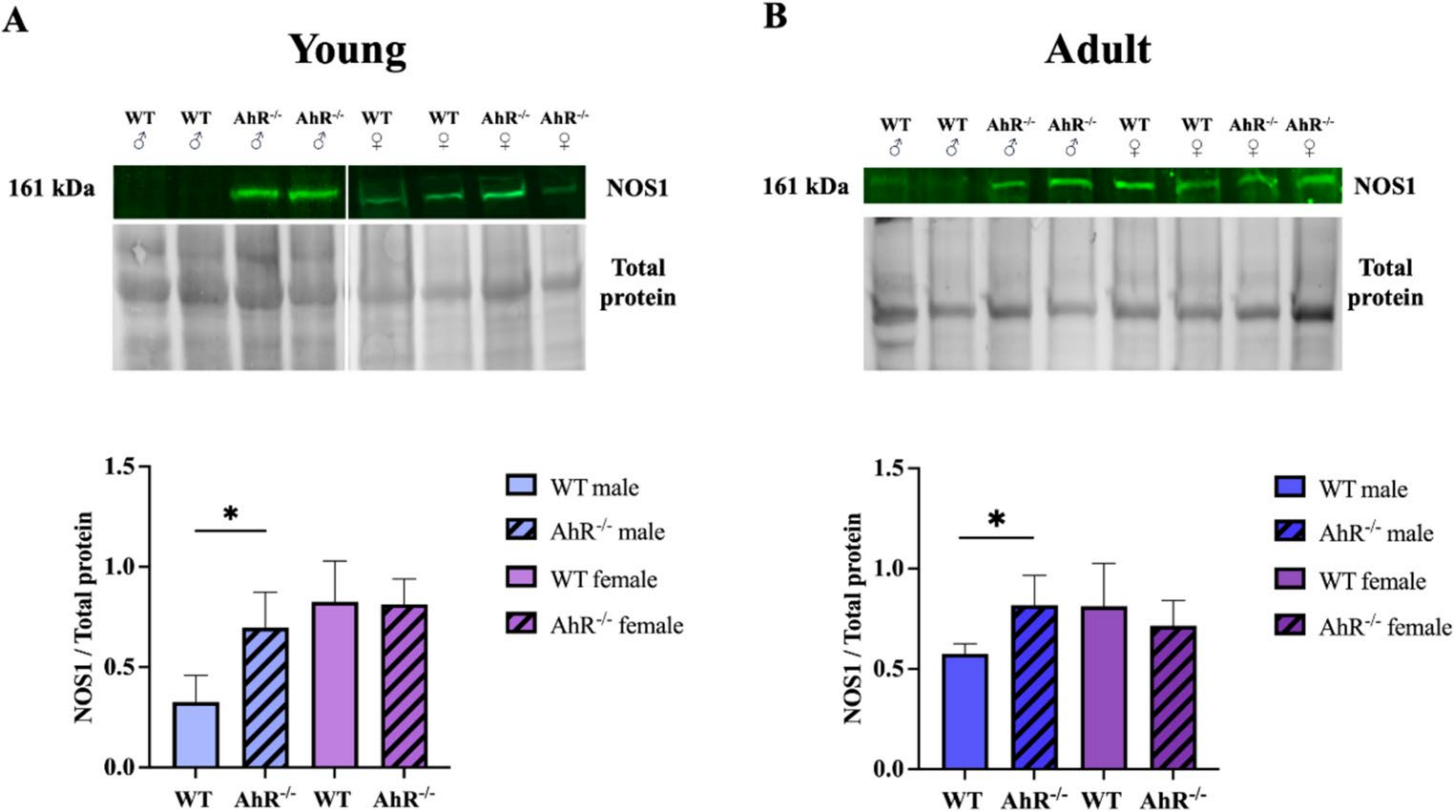
NOS1 protein expression in colon. The figure shows representative immunoblots and NOS1 protein expression in WT and AhR^−/−^ male and female mice in young (**A**) and adult (**B**) groups. Densitometric values were normalized to total protein using Stain-Free technology. Data are expressed as mean ± SD and were analyzed using two-way ANOVA followed by a Bonferroni post hoc test (*n* = 4 per group). *p < 0.05 vs. WT male

A decrease in PGP9.5 protein expression was observed in young AhR^−/−^ females compared with WT females (Fig. 13A). No differences were detected in the remaining experimental groups (Fig. 13A, B). Moreover, NOS1 protein expression increased in both young and adult AhR^−/−^ males when compared to their WT littermates with no differences in females (Fig. 14).

### Gene expression study

**Fig. 15:**
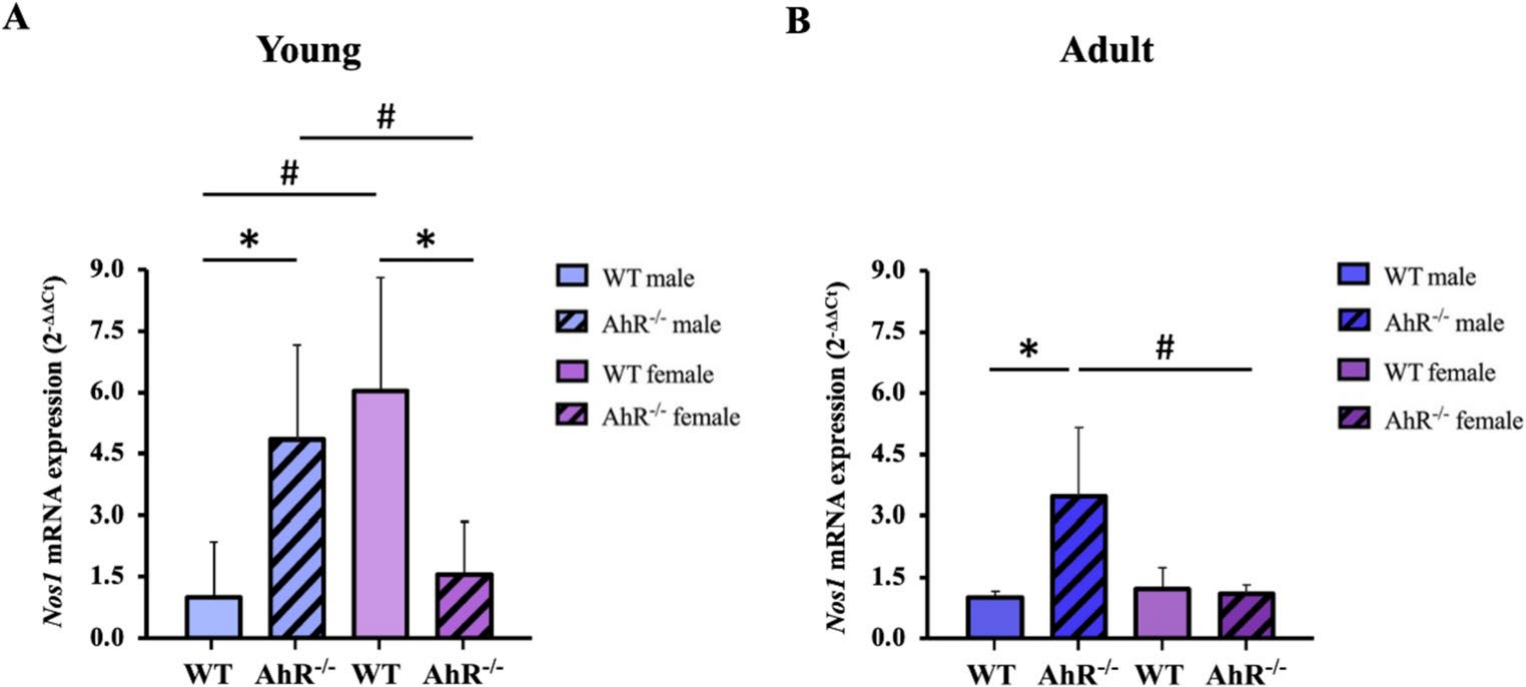
Relative gene expression of *Nos1* in colon. The figure shows the mRNA relative expression of the *Nos1* in the colon of WT and AhR^−/−^ in young (**A**) and adult (**B**) mice. Data are expressed as mean ± SEM and were analyzed using two-way ANOVA followed by a Bonferroni post hoc test (*n* = 6–7 per group). *p < 0.05 vs. the same-sex WT group; ^#^*p* < 0.05 vs. male of the same genotype

*Nos1* mRNA expression was increased in colon of young and adult AhR^−/−^ males (Fig. 15A, B). By contrast, the expression level of *Nos1* mRNA was reduced in young AhR^−/−^ females in comparison with WT females, while no alterations were observed in adult subjects (Fig. 15A, B). A significant increase in expression was observed in young WT females compared to WT males (Fig. 15A). But in young and adult mice, levels of *Nos1* were reduced in AhR^−/−^ females in comparison to AhR^−/−^ males (Fig. 15B).

**Fig. 16:**
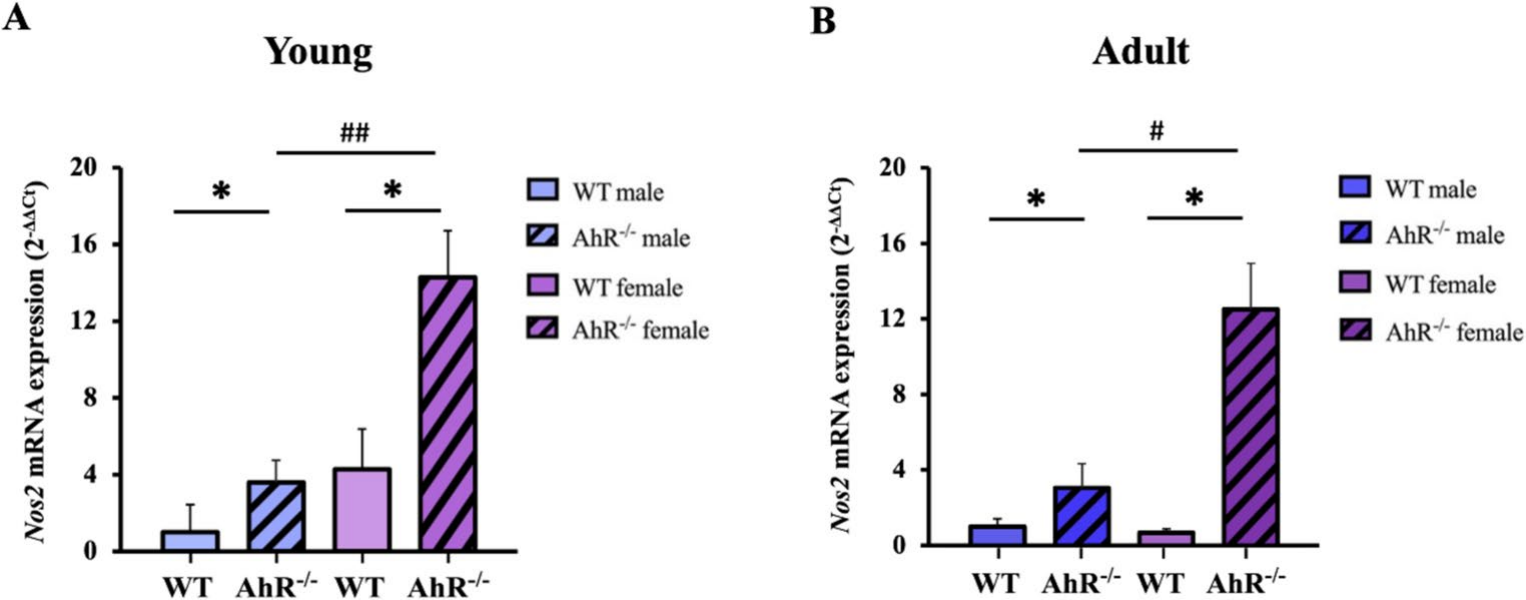
Relative gene expression of *Nos2* in colon. The figure shows the mRNA relative expression of the *Nos2* in the colon of WT and AhR^−/−^ in young (**A**) and adult (**B**) mice. Data are expressed as mean ± SEM and were analyzed using two-way ANOVA followed by a Bonferroni post hoc test (*n* = 6–7 per group). *p < 0.05 vs. the same-sex WT group; ^#^*p* < 0.05, ^##^*p* < 0.01 vs. AhR^−/−^ male

*Nos2* gene expression was increased in both sexes in young (Fig. 16A) and adult (Fig. 16B) from AhR^−/−^ in comparison with WT mice. In young and adult mice, expression in AhR^−/−^ females was higher than in males (Fig. 16A, B).

**Fig. 17:**
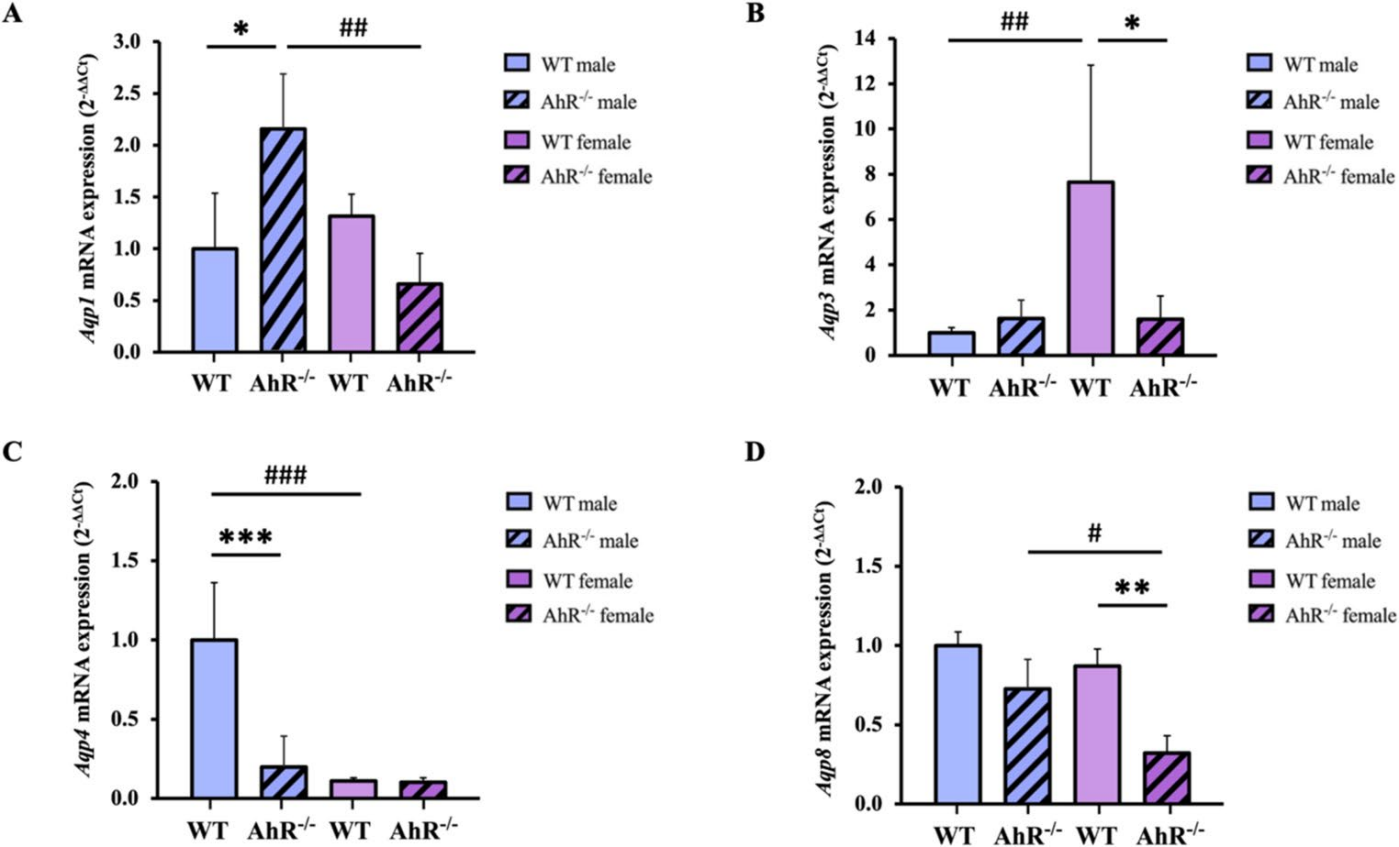
Relative gene expression of *Aqp1*, *Aqp3*, *Aqp4* and *Aqp8* in colon from young mice. The figure shows the relative expression in the colon of WT and AhR^−/−^ young mice of *Aqp1* (**A**), *Aqp3* (**B**), *Aqp4* (**C**), and *Aqp8* (**D**). Data are expressed as mean ± SEM and data were analyzed using two-way ANOVA followed by a Bonferroni post hoc test (*n* = 6–7 per group). *p < 0.05, **p < 0.01, ***p < 0.001 vs. the same-sex WT group; ^#^*p* < 0.05, ^##^*p* < 0.01, ^###^*p* < 0.001 vs. male of the same genotype

**Fig. 18:**
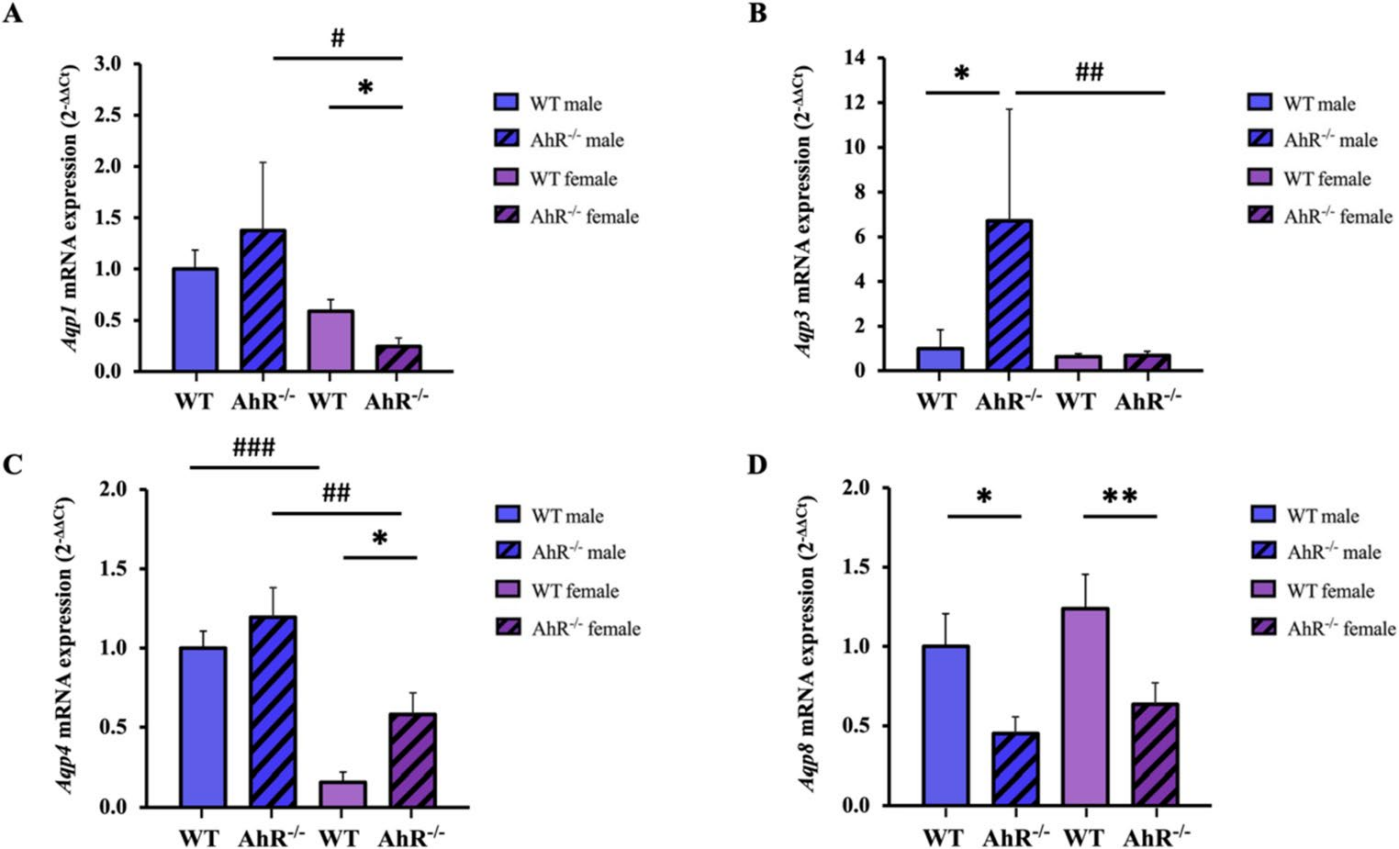
Relative gene expression of *Aqp1*, *Aqp3*, *Aqp4* and *Aqp8* in colon from adult mice. The figure shows the relative expression in the colon of WT and AhR^−/−^ adult mice of *Aqp1* (**A**), *Aqp3* (**B**), *Aqp4* (**C**), and *Aqp8* (**D**). Data are expressed as mean ± SEM and data were analyzed using two-way ANOVA followed by a Bonferroni post hoc test (*n* = 6–7 per group). *p < 0.05, **p < 0.01 vs. the same-sex WT group; ^#^*p* < 0.05, ^##^*p* < 0.01, ^###^*p* < 0.001 vs. male of the same genotype

Aquaporin expression exhibited sex- and age-dependent deregulation (Figs. 17 and 18).

In young AhR^−/−^ males, *Aqp1* expression increased, while *Aqp4* expression decreased (Fig. 17A, C). In contrast, adult AhR^−/−^ males exhibited an upregulation of *Aqp3* and a downregulation of *Aqp8* (Fig. 18B, D).

In the case of female mice, *Aqp3* and *Aqp8* expression was concurrently decreased in young AhR^−/−^ individuals (see Fig. 17B, D). However, during adulthood, AhR^−/−^ females exhibited an upregulation of *Aqp4*, alongside a downregulation of both *Aqp1* and *Aqp8* (Fig. 18A, C, D).

When comparing sex within the same genotype, young AhR^−/−^ females, exhibited a decreased expression of *Aqp1* and *Aqp8* relative to AhR^−/−^ males, whereas *Aqp3* was increased in WT females and *Aqp4* decreased when compared to WT males (Fig. 17A-D). In adult AhR^−/−^ females, *Aqp1*, *Aqp3*, and *Aqp4* expression levels were reduced in comparison to those observed in AhR^−/−^ males, with an additional decrease in *Aqp4* observed in WT females (Fig. 18A-C).

**Fig. 19:**
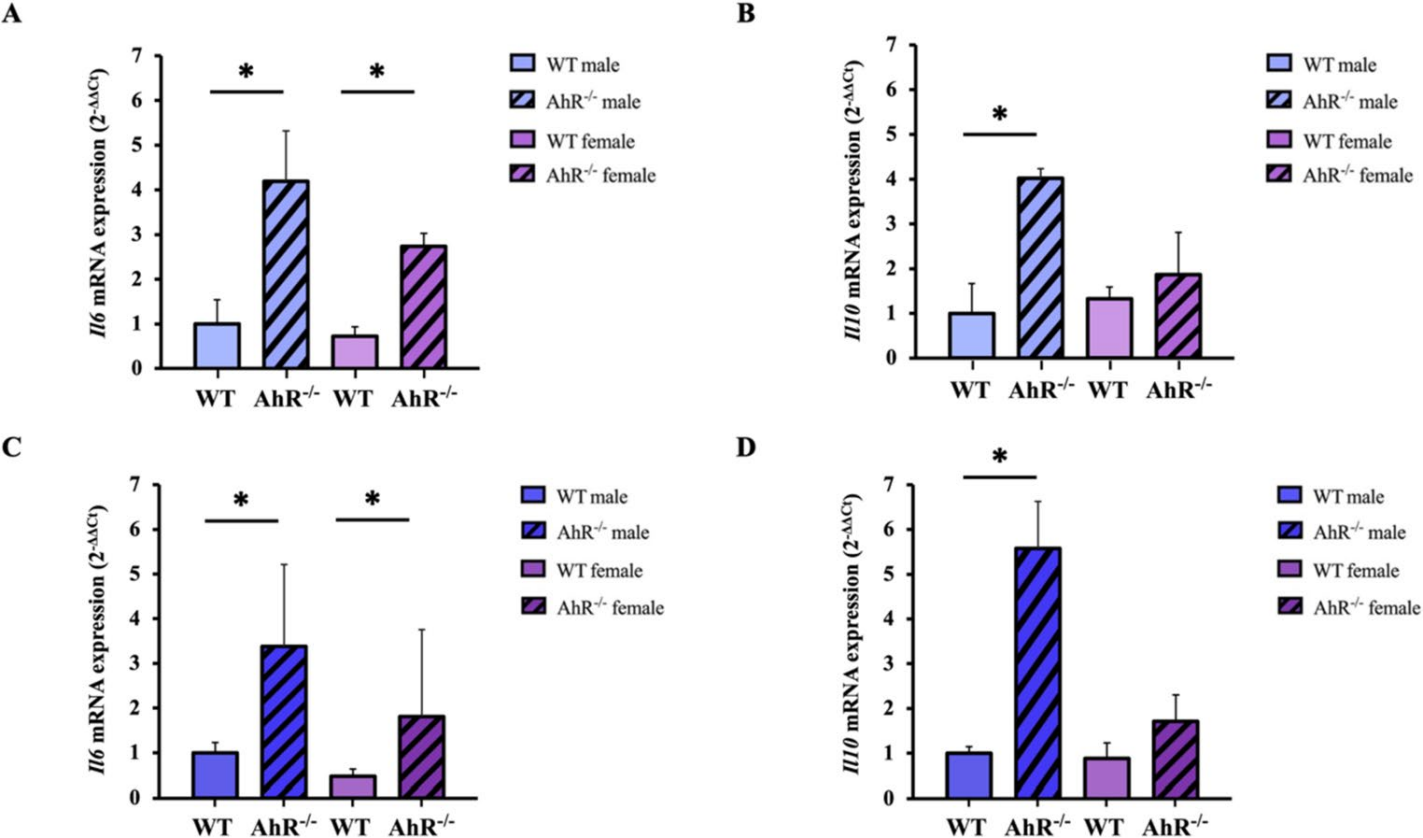
Relative gene expression of *Il6* and *Il10* in colon from young and adult mice. The figure shows the relative expression of *Il6* (**A**) and *Il10* (**B**) in the colon of WT and AhR^−/−^ young mice and of *Il6* (**C**) and *Il10* (**D**) in the colon of WT and AhR^−/−^ adult mice. Data are expressed as mean ± SEM (*n* = 3 per group) and were analyzed using the non-parametric Dunn’s test. *p < 0.05 vs. the same-sex WT group

The expression of interleukins 6 (*Il6*) and 10 (*Il10*) was evaluated as markers of the inflammatory status of mouse colonic tissue. In AhR^−/−^ males, *Il6* and *Il10* expression increased at both ages (Fig. 19A - D). In females, *Il6* expression was increased in young and adult AhR^−/−^ mice (Fig. 19A, C) in comparison with WT females, whereas *Il10* expression showed no changes (Fig. 19B, D).

## Discussion

AhR deficiency has been previously associated with reduced lifespan and accelerated aging phenotypes. In our previous work, including studies in *Caenorhabditis elegans*, we have demonstrated that the AhR ortholog modulates lifespan, stress resistance, and healthspan, supporting a conserved role for AhR in the regulation of organismal homeostasis [24]. Consistent with previous reports, our survival curves confirmed that AhR deficiency is associated with reduced survival and accelerated mortality in both male and female mice. In parallel, aging is associated with an increased burden of gastrointestinal disorders and a progressive decline in gastrointestinal function, contributing to the higher prevalence of constipation in older individuals [25]. Proposed mechanisms include alterations in enteric nervous system and age-related shifts in gut microbiota composition [15, 26].

In this context, AhR has emerged as a key regulator of intestinal homeostasis [27, 28]. Previous studies have examined the role of AhR in the enteric nervous system, focusing primarily on its activation by toxic compounds such as 2,3,7,8-tetrachlorodibenzo-p-dioxin (TCDD) [18]. However, because AhR is physiologically activated by a wide range of endogenous, dietary, and microbiota-derived ligands, the effects induced by xenobiotic activation may not fully reflect its normal biological functions. Consequently, the specific contribution of AhR to enteric motor regulation during aging remains incompletely understood. Here, we provide an integrated phenotypic and functional analysis of AhR-deficient mice, identifying a predominantly female colonic motor phenotype associated with early neuromuscular and inflammatory alterations.

A major finding of this study is the selective reduction in spontaneous colonic peristalsis in AhR^−/−^ females at both young and adult ages, while males remain largely unaffected. This functional impairment was accompanied by reduced stool number observed in AhR^−/−^ females, suggesting that loss of AhR compromises basal propulsive activity and/or colonic motor patterns in a sex-specific manner. Furthermore, structural alterations were also detected in young AhR^−/−^ females, which exhibited reduced thickness of both circular and longitudinal muscle layers. These changes were not observed in males or in aging, suggesting that AhR deficiency induces early alterations in colonic function and structure that may later be compensated by other mechanisms over time. Consistent with these findings, PGP9.5 protein expression and ACh-induced contraction were diminished exclusively in young AhR^−/−^ females. Since PGP9.5 is a broad neuronal marker [29], this reduction should not be interpreted as evidence of a specific neuronal subtype loss or as a direct measure of neuronal density. Rather, it indicates an alteration in the enteric neuronal compartment or in the abundance of neuronal-associated proteins during early life. Thus, reduced PGP9.5 expression suggests that AhR deficiency may alter the enteric neuronal compartment or neuromuscular regulation in young females, potentially contributing to impaired spontaneous motor activity.

Our study also suggests altered nitrergic modulation in the colon of AhR-deficient mice only in males. In WT males, inhibition of nitric oxide synthase with L-NAME increased spontaneous peristaltic amplitude, consistent with tonic nitrergic inhibitory control. This response was absent in AhR^−/−^ males, indicating altered or impaired nitrergic modulation. In contrast, L-NAME did not significantly modify spontaneous peristalsis in females of either genotype, suggesting a limited contribution of nitrergic inhibition under our experimental conditions.

Interestingly, this functional impairment occurred despite increased *Nos1* expression in AhR^−/−^ males, indicating that the observed increase in gene and protein expression is probably a compensatory response to impaired function.

AhR deficiency was also associated with a marked upregulation of *Nos2* gene expression, particularly in females at both age. Inducible NOS is typically associated with inflammatory conditions and may disrupt physiological neuromuscular signaling through sustained nitric oxide (NO) production and nitrosative stress [30]. Consistent with this finding, *Il6* expression was increased in AhR^−/−^ mice of both sexes and at both ages, supporting the presence of a pro-inflammatory colonic environment. IL-6 is a key mediator of intestinal inflammation and has been associated with altered enteric neuronal function and disturbed colonic motility [31, 32]. In contrast, *Il10* expression was increased in AhR^−/−^ males at both ages, whereas no such increase was observed in females. Given the well-established anti-inflammatory role of IL-10, this finding may indicate the activation of compensatory regulatory mechanisms in males that partially counterbalance the inflammatory consequences of AhR deficiency [33]. Nevertheless, these findings should be interpreted cautiously given the limited sample size of the cytokine analyses.

In parallel, AhR deficiency also induced sex- and age-dependent changes in aquaporin expression, although no consistent pattern emerged across the different isoforms. Aquaporins contribute to epithelial water transport in the colon and can be modulated by inflammatory mediators and bile acids [34]. However, fecal water content remained unchanged across sexes, ages, and genotypes, suggesting that these transcriptional changes are unlikely to be major determinants of stool hydration or motility alterations in this model. Interestingly, AhR^−/−^ females consistently exhibited lighter stool coloration at both ages. Although the underlying mechanism remains unclear, stool color is influenced by luminal factors such as microbial metabolism and bile pigment processing, both of which may be affected by AhR signaling. Therefore, this finding may reflect broader changes in the intestinal environment associated with AhR deficiency and warrants further investigation.

This study has several limitations. We did not directly quantify enteric neuronal subtypes (e.g., nitrergic neurons by immunohistochemistry), nor did we measure NO release or electrophysiological patterns that could more precisely define alterations in inhibitory motor pathways. In addition, microbiota composition and luminal metabolite profiles were not evaluated, despite their central role in AhR signaling. Finally, the adult age examined corresponds to mid-life rather than advanced age in mice, and future studies at later ages will be necessary to determine how AhR-dependent dysmotility progresses during aging.

Sex differences were evident throughout the study, particularly in the severity of the colonic motor phenotype observed in young females. These differences, together with the alterations in spontaneous peristalsis and PGP9.5 protein expression, suggest that female-specific biological factors may contribute to the observed phenotype. Previous studies have described interactions between AhR signaling and estrogen-related pathways [35] In addition, estrogen receptors are expressed in the gastrointestinal tract, and estrogens have been implicated in the regulation of intestinal motility [36–39]. However, hormonal parameters were not assessed in the present study, and therefore any contribution of estrogen signaling to the observed phenotype remains speculative and requires further investigation.

The mechanisms underlying the age-dependent differences observed in this study remain unclear. One possibility is that age-related changes in AhR signaling pathways may modify the physiological consequences of receptor deficiency over time. Further studies will be required to determine whether differential engagement of canonical and non-canonical AhR signaling contributes to these age-dependent effects.

In conclusion, AhR deficiency induces a sex-dependent colonic motor phenotype that predominantly affects females, combining functional impairment with early neuromuscular remodeling. In contrast, AhR deficiency in males primarily alters nitrergic modulation without overt motor dysfunction. In addition, AhR deficiency is associated with a pro-inflammatory colonic environment characterized by increased *Nos2* and *Il6* expression. Collectively, these findings support a role for AhR in enteric neuromuscular regulation and intestinal homeostasis and provide new insights into the mechanisms underlying age-related intestinal dysfunction. Furthermore, our findings raise the possibility that AhR signaling could be targeted through dietary or microbiota-derived ligands to modulate intestinal function and promote gastrointestinal health.

## Electronic Supplementary Material

Below is the link to the electronic supplementary material.

Supplementary file 1

Supplementary file 2 Supplementary Figure S1. Representative images of 24-h fecal output from young male and female in WT and AhR-/- mice under standardized imaging conditions. Supplementary Figure S2. Representative images of 24-h fecal output from adult male and female in WT and AhR-/- mice under standardized imaging conditions. Supplementary Figure S3. Spontaneous peristalsis. Frequency (Hz) was measured in 1-cm-long colon segments of WT and AhR-/- male and female mice in young (A) and adult (B) groups. Data are expressed as mean ± SEM and were analyzed using two-way ANOVA followed by a Bonferroni post hoc test (n = 5 – 15 per group).

## Data Availability

Data are contained within this article.

## References

[1] Beischlag TV, Luis Morales J, Hollingshead BD, Perdew GH (2008) The aryl hydrocarbon receptor complex and the control of gene expression. Crit Rev Eukaryot Gene Expr 18(3):207–250. 10.1615/critreveukargeneexpr.v18.i3.20 PubMed PMID: 18540824; PubMed Central PMCID: PMC2583464 18540824 10.1615/critreveukargeneexpr.v18.i3.20 PMC2583464

[2] Kou Z, Dai W (2021) Aryl hydrocarbon receptor: Its roles in physiology. Biochem Pharmacol 185:114428. 10.1016/j.bcp.2021.114428 PubMed PMID: 33515530; PubMed Central PMCID: PMC8862184 33515530 10.1016/j.bcp.2021.114428 PMC8862184

[3] Lahvis GP, Pyzalski RW, Glover E, Pitot HC, McElwee MK, Bradfield CA (2005) The aryl hydrocarbon receptor is required for developmental closure of the ductus venosus in the neonatal mouse. Mol Pharmacol 67(3):714–720. 10.1124/mol.104.008888 PubMed PMID: 15590894 15590894 10.1124/mol.104.008888

[4] Lamas B, Natividad JM, Sokol H (2018) Aryl hydrocarbon receptor and intestinal immunity. Mucosal Immunol 11(4):1024–1038. 10.1038/s41385-018-0019-2 PubMed PMID: 29626198 29626198 10.1038/s41385-018-0019-2

[5] Yue B, Yu ZL, Lv C, Geng XL, Wang ZT, Dou W (2020) Regulation of the intestinal microbiota: An emerging therapeutic strategy for inflammatory bowel disease. World J Gastroenterol 26(30):4378–4393. 10.3748/wjg.v26.i30.4378 PubMed PMID: 32874052; PubMed Central PMCID: PMC7438192 32874052 10.3748/wjg.v26.i30.4378 PMC7438192

[6] Li Y, Innocentin S, Withers DR, Roberts NA, Gallagher AR, Grigorieva EF et al (2011) Exogenous stimuli maintain intraepithelial lymphocytes via aryl hydrocarbon receptor activation. Cell 147(3):629–640. 10.1016/j.cell2011.09.025 PubMed PMID: 21999944 21999944 10.1016/j.cell.2011.09.025

[7] de Vos WM, Tilg H, Van Hul M, Cani PD (2022) Gut microbiome and health: mechanistic insights. Gut 71(5):1020–1032. 10.1136/gutjnl-2021-326789 PubMed PMID: 35105664; PubMed Central PMCID: PMC8995832 35105664 10.1136/gutjnl-2021-326789 PMC8995832

[8] Burton KJ, Pimentel G, Zangger N, Vionnet N, Drai J, McTernan PG et al (2018) Modulation of the peripheral blood transcriptome by the ingestion of probiotic yoghurt and acidified milk in healthy, young men. PLoS ONE 13(2):e0192947. 10.1371/journal.pone.0192947 PubMed PMID: 29489876; PubMed Central PMCID: PMC5831037 29489876 10.1371/journal.pone.0192947 PMC5831037

[9] Lamas B, Richard ML, Leducq V, Pham HP, Michel ML, Da Costa G et al (2016) CARD9 impacts colitis by altering gut microbiota metabolism of tryptophan into aryl hydrocarbon receptor ligands. Nat Med 22(6):598–605. 10.1038/nm.4102 PubMed PMID: 27158904; PubMed Central PMCID: PMC5087285 27158904 10.1038/nm.4102 PMC5087285

[10] Nolan LS, Mihi B, Agrawal P, Gong Q, Rimer JM, Bidani SS et al (2021) Indole-3-Carbinol-Dependent Aryl Hydrocarbon Receptor Signaling Attenuates the Inflammatory Response in Experimental Necrotizing Enterocolitis. ImmunoHorizons 5(4):193–209. 10.4049/immunohorizons.2100018 PubMed PMID: 33906960; PubMed Central PMCID: PMC8173979 33906960 10.4049/immunohorizons.2100018 PMC8173979

[11] Parlesak A, Klein B, Schecher K, Bode JC, Bode C (2003) Prevalence of small bowel bacterial overgrowth and its association with nutrition intake in nonhospitalized older adults. J Am Geriatr Soc 51(6):768–773. 10.1046/j.1365-2389.2003.51259.x PubMed PMID: 12757562 12757562 10.1046/j.1365-2389.2003.51259.x

[12] Ke S, Mitchell SJ, MacArthur MR, Kane AE, Sinclair DA, Venable EM et al (2021) Gut Microbiota Predicts Healthy Late-Life Aging in Male Mice. Nutrients 13(9):3290. 10.3390/nu13093290 PubMed PMID: 34579167; PubMed Central PMCID: PMC8467910 34579167 10.3390/nu13093290 PMC8467910

[13] Bernard CE, Gibbons SJ, Gomez-Pinilla PJ, Lurken MS, Schmalz PF, Roeder JL et al (2009) Effect of age on the enteric nervous system of the human colon. Neurogastroenterol Motil 21(7):746–e46. 10.1111/j.1365-2982.2008.01245x PubMed PMID: 19220755; PubMed Central PMCID: PMC2776702 19220755 10.1111/j.1365-2982.2008.01245.x PMC2776702

[14] Phillips RJ, Pairitz JC, Powley TL (2007) Age-related neuronal loss in the submucosal plexus of the colon of Fischer 344 rats. Neurobiol Aging 28(7):1124–1137. 10.1016/j.neurobiolaging.200605.019 PubMed PMID: 16793176 16793176 10.1016/j.neurobiolaging.2006.05.019

[15] Dumic I, Nordin T, Jecmenica M, Stojkovic Lalosevic M, Milosavljevic T, Milovanovic T (2019) Gastrointestinal Tract Disorders in Older Age. Can J Gastroenterol Hepatol 2019:6757524. 10.1155/2019/6757524 PubMed PMID: 30792972; PubMed Central PMCID: PMC6354172 30792972 10.1155/2019/6757524 PMC6354172

[16] Serna E, Cespedes C, Vina J (2020) Anti-Aging Physiological Roles of Aryl Hydrocarbon Receptor and Its Dietary Regulators. Int J Mol Sci 22(1):374. 10.3390/ijms22010374 PubMed PMID: 33396477; PubMed Central PMCID: PMC7795126 33396477 10.3390/ijms22010374 PMC7795126

[17] Bravo-Ferrer I, Cuartero MI, Medina V, Ahedo-Quero D, Peña-Martínez C, Pérez-Ruíz A et al (2019) Lack of the aryl hydrocarbon receptor accelerates aging in mice. FASEB J Off Publ Fed Am Soc Exp Biol 33(11):12644–12654. 10.1096/fj.201901333R PubMed PMID: 31483997 10.1096/fj.201901333R 31483997

[18] Vijay A, Boyle NR, Kumar SM, Perdew GH, Srinivasan S, Patterson AD (2023) Aryl hydrocarbon receptor activation affects nitrergic neuronal survival and delays intestinal motility in mice. Toxicol Sci 192(1):117–128. 10.1093/toxsci/kfad014 36782369 10.1093/toxsci/kfad014 PMC10025877

[19] Lee J, Prokopec SD, Watson JD, Sun RX, Pohjanvirta R, Boutros PC (2015) Male and female mice show significant differences in hepatic transcriptomic response to 2,3,7,8-tetrachlorodibenzo-p-dioxin. BMC Genomics 16(1):625. 10.1186/s12864-015-1840-6 PubMed PMID: 26290441; PubMed Central PMCID: PMC4546048 26290441 10.1186/s12864-015-1840-6 PMC4546048

[20] Brierley SM, Nichols K, Grasby DJ, Waterman SA (2001) Neural mechanisms underlying migrating motor complex formation in mouse isolated colon. Br J Pharmacol 132(2):507–517. 10.1038/sj.bjp.0703814 PubMed PMID: 11159701; PubMed Central PMCID: PMC1572567 11159701 10.1038/sj.bjp.0703814 PMC1572567

[21] Kerr MM, Gourlay T (2021) Design and numerical simulation for the development of an expandable paediatric heart valve. Int J Artif Organs 44(7):518–524 doi:10.1177/0391398820977509 PubMed PMID: 33300423; PubMed Central PMCID: PMC8366171 33300423 10.1177/0391398820977509 PMC8366171

[22] Gilda JE, Gomes AV (2013) Stain-Free total protein staining is a superior loading control to β-actin for Western blots. Anal Biochem 440(2):186–188. 10.1016/j.ab.2013.05.027 23747530 10.1016/j.ab.2013.05.027 PMC3809032

[23] Livak KJ, Schmittgen TD (2001) Analysis of relative gene expression data using real-time quantitative PCR and the 2(-Delta Delta C(T)) Method. Methods 25(4):402–408. 10.1006/meth.2001.1262 PubMed PMID: 11846609 11846609 10.1006/meth.2001.1262

[24] Serna E, Verdú D, Valls A, Belenguer-Varea Á, Tarazona-Santabalbina FJ, Borrás C et al (2024) Involvement of aryl hydrocarbon receptor in longevity and healthspan: insights from humans, mice, and C. elegans. Int J Mol Sci 25(18):9943. 10.3390/ijms25189943 39337431 10.3390/ijms25189943 PMC11432571

[25] Tran L, Greenwood-Van Meerveld B (2014) In a non-human primate model, aging disrupts the neural control of intestinal smooth muscle contractility in a region-specific manner. Neurogastroenterol Motil 26(3):410–418. 10.1111/nmo.12290 PubMed PMID: 24548258; PubMed Central PMCID: PMC4077178 24548258 10.1111/nmo.12290 PMC4077178

[26] Sun T, Li D, Hu S, Huang L, Sun H, Yang S et al (2018) Aging-dependent decrease in the numbers of enteric neurons, interstitial cells of Cajal and expression of connexin43 in various regions of gastrointestinal tract. Aging 10(12):3851–3865. 10.18632/aging.101677 PubMed PMID: 30530917; PubMed Central PMCID: PMC6326649 30530917 10.18632/aging.101677 PMC6326649

[27] Roager HM, Licht TR (2018) Microbial tryptophan catabolites in health and disease. Nat Commun 9(1):3294. 10.1038/s41467018-05470-4 PubMed PMID: 30120222; PubMed Central PMCID: PMC6098093 30120222 10.1038/s41467-018-05470-4 PMC6098093

[28] Dong F, Perdew GH (2020) The aryl hydrocarbon receptor as a mediator of host-microbiota interplay. Gut Microbes 12(1):1859812. 101080/19490976.2020.1859812 PubMed PMID: 33382356; PubMed Central PMCID: PMC7781536 33382356 10.1080/19490976.2020.1859812 PMC7781536

[29] Guerra DD, Bok R, Vyas V, Orlicky DJ, Lorca RA, Hurt KJ (2019) Akt phosphorylation of neuronal nitric oxide synthase regulates gastrointestinal motility in mouse ileum. Proc Natl Acad Sci U S A 116(35):17541–17546. 10.1073/pnas.1905902116 PubMed PMID: 31405982; PubMed Central PMCID: PMC6717252 31405982 10.1073/pnas.1905902116 PMC6717252

[30] Mahavadi S, Nalli AD, Kumar DP, Hu W, Kuemmerle JF, Grider JR et al (2014) Cytokine-induced iNOS and ERK1/2 inhibit adenylyl cyclase type 5/6 activity and stimulate phosphodiesterase 4D5 activity in intestinal longitudinal smooth muscle. Am J Physiol Cell Physiol 307(4):C402–411. 10.1152/ajpcell.00123.2014 PubMed PMID: 24944202; PubMed Central PMCID: PMC4137135 24944202 10.1152/ajpcell.00123.2014 PMC4137135

[31] Naito Y, Takagi T, Uchiyama K, Kuroda M, Kokura S, Ichikawa H et al (2004) Reduced intestinal inflammation induced by dextran sodium sulfate in interleukin-6-deficient mice. Int J Mol Med 14(2):191–196 PubMed PMID: 15254764 15254764

[32] Buckley MM, O’Halloran KD, Rae MG, Dinan TG, O’Malley D (2014) Modulation of enteric neurons by interleukin-6 and corticotropin-releasing factor contributes to visceral hypersensitivity and altered colonic motility in a rat model of irritable bowel syndrome. J Physiol 592(23):5235–5250. 10.1113/jphysiol.2014.279968 PubMed PMID: 25260633; PubMed Central PMCID: PMC4262336 25260633 10.1113/jphysiol.2014.279968 PMC4262336

[33] Meador BM, Krzyszton CP, Johnson RW, Huey KA (2008) Effects of IL-10 and age on IL-6, IL-1beta, and TNF-alpha responses in mouse skeletal and cardiac muscle to an acute inflammatory insult. J Appl Physiol 104(4):991–997. 10.1152/japplphysiol.01079.2007 PubMed PMID: 18218915 18218915 10.1152/japplphysiol.01079.2007

[34] Yde J, Keely S, Wu Q, Borg JF, Lajczak N, O’Dwyer A et al (2016) Characterization of AQPs in Mouse, Rat, and Human Colon and Their Selective Regulation by Bile Acids. Front Nutr 3:46. 10.3389/fnut.2016.00046 PubMed PMID: 27777930; PubMed Central PMCID: PMC5056181 27777930 10.3389/fnut.2016.00046 PMC5056181

[35] Barnett KR, Tomic D, Gupta RK, Miller KP, Meachum S, Paulose T et al (2007) The aryl hydrocarbon receptor affects mouse ovarian follicle growth via mechanisms involving estradiol regulation and responsiveness. Biol Reprod 76(6):1062–1070. 10.1095/biolreprod.106.057687 PubMed PMID: 17329597 17329597 10.1095/biolreprod.106.057687

[36] Kawano N, Koji T, Hishikawa Y, Murase K, Murata I, Kohno S (2004) Identification and localization of estrogen receptor alpha- and beta-positive cells in adult male and female mouse intestine at various estrogen levels. Histochem Cell Biol 121(5):399–405. 10.1007/s00418-004-0644-6 PubMed PMID: 15138841 15138841 10.1007/s00418-004-0644-6

[37] Li Y, Xu J, Jiang F, Jiang Z, Liu C, Li L et al (2016) G protein-coupled estrogen receptor is involved in modulating colonic motor function via nitric oxide release in C57BL/6 female mice. Neurogastroenterol Motil 28(3):432–442. 10.1111/nmo.12743 PubMed PMID: 26661936 26661936 10.1111/nmo.12743

[38] Zielińska M, Fichna J, Bashashati M, Habibi S, Sibaev A, Timmermans JP et al (2017) G protein-coupled estrogen receptor and estrogen receptor ligands regulate colonic motility and visceral pain. Neurogastroenterol Motil 29(7). 10.1111/nmo.13025 PubMed PMID: 28191706 10.1111/nmo.13025 28191706

[39] Ravella K, Al-Hendy A, Sharan C, Hale AB, Channon KM, Srinivasan S et al (2013) Chronic estrogen deficiency causes gastroparesis by altering neuronal nitric oxide synthase function. Dig Dis Sci 58(6):1507–1515. 10.1007/s10620-013-2610-4 PubMed PMID: 23504347; PubMed Central PMCID: PMC3691310 23504347 10.1007/s10620-013-2610-4 PMC3691310

