# Supplementary material for: Aryl hydrocarbon receptor deficiency leads to sex- and age-dependent colonic dysmotility in mice
Source: J Physiol Biochem. 2026 Jul 16;82(1):71. doi: 10.1007/s13105-026-01208-9 (PMC13375827; doi:10.1007/s13105-026-01208-9)

Complete Blots for figure 13A


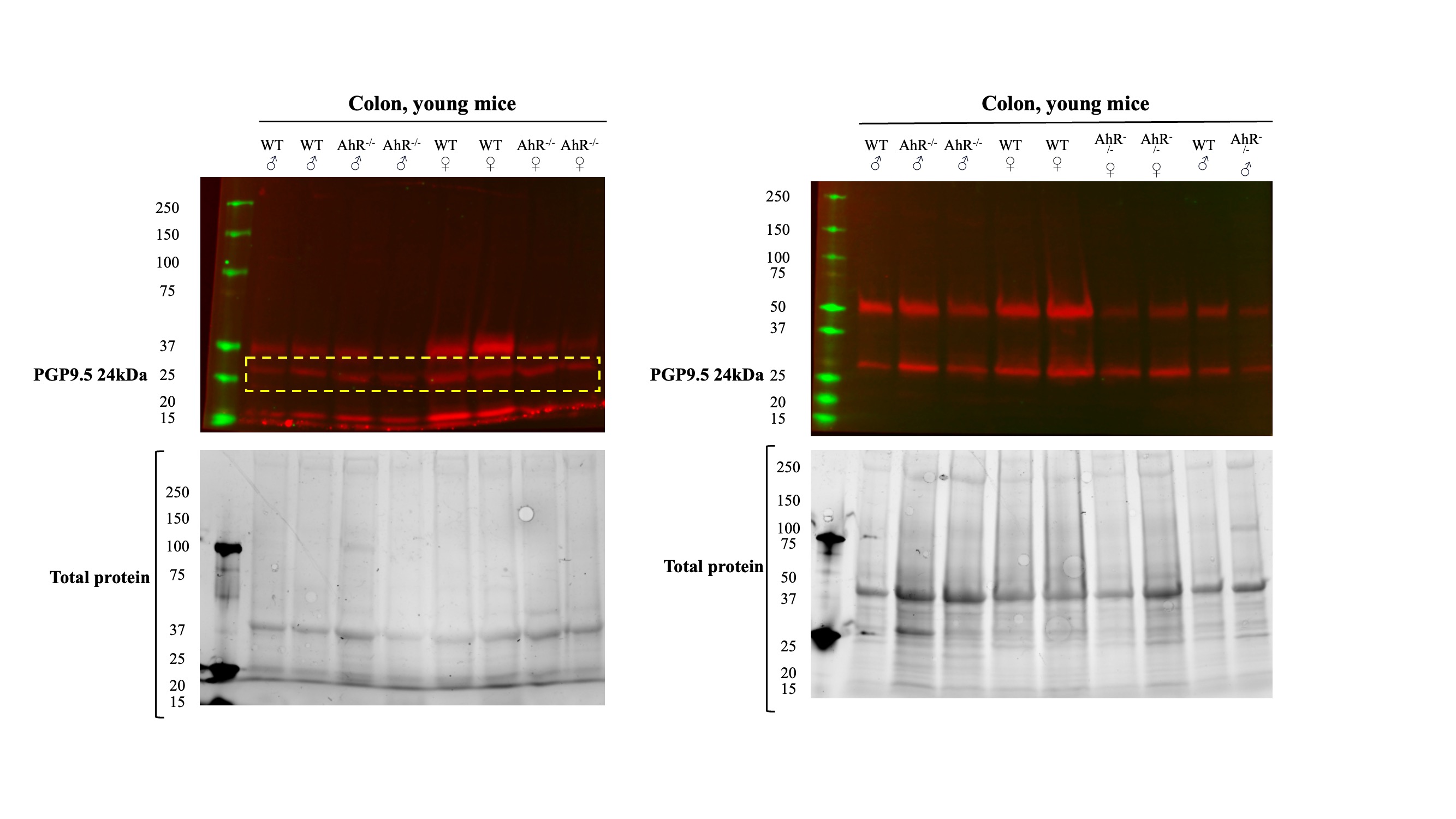

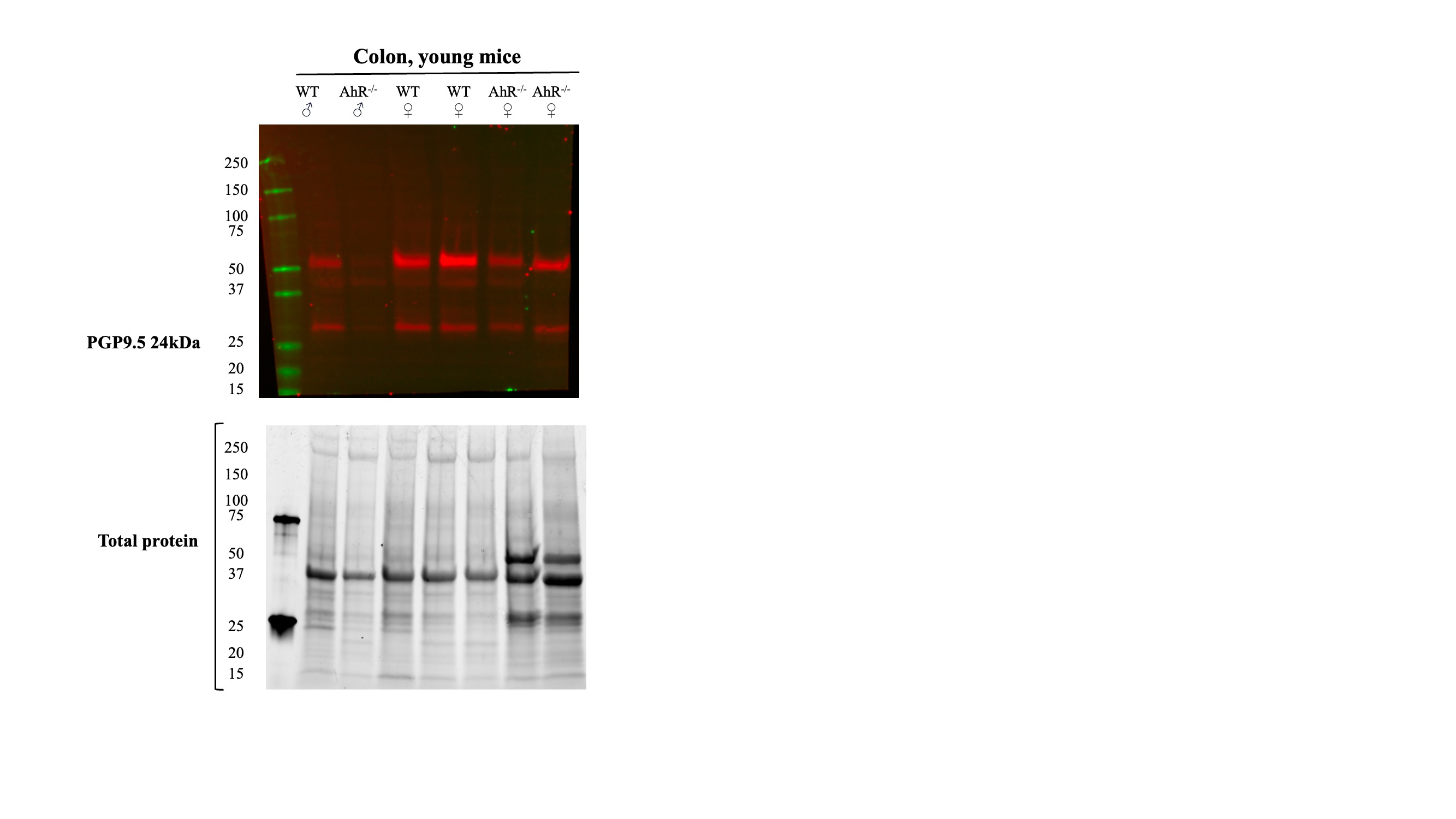


Complete Blots for figure 13B


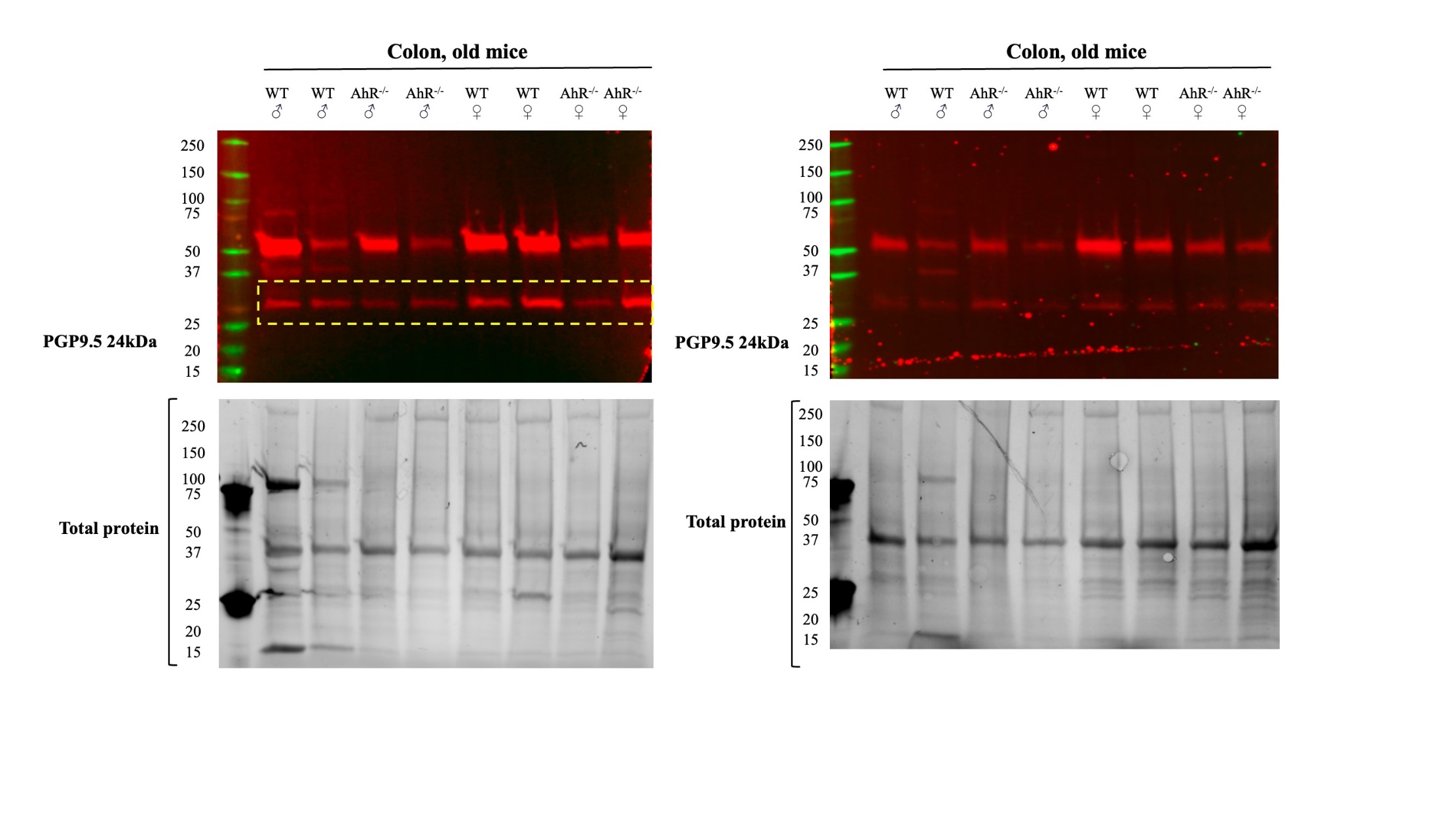

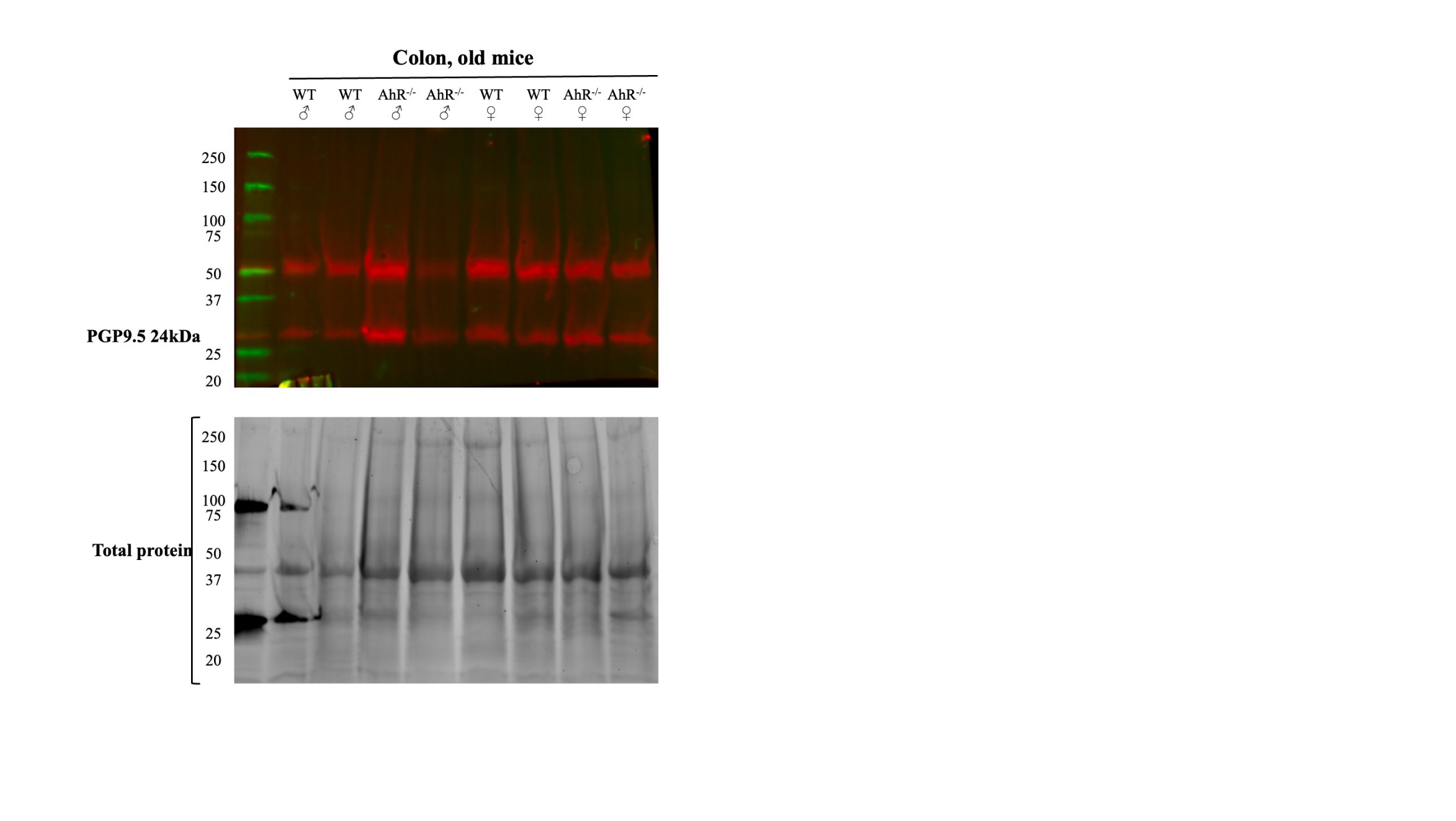


Complete Blots for figure 14A


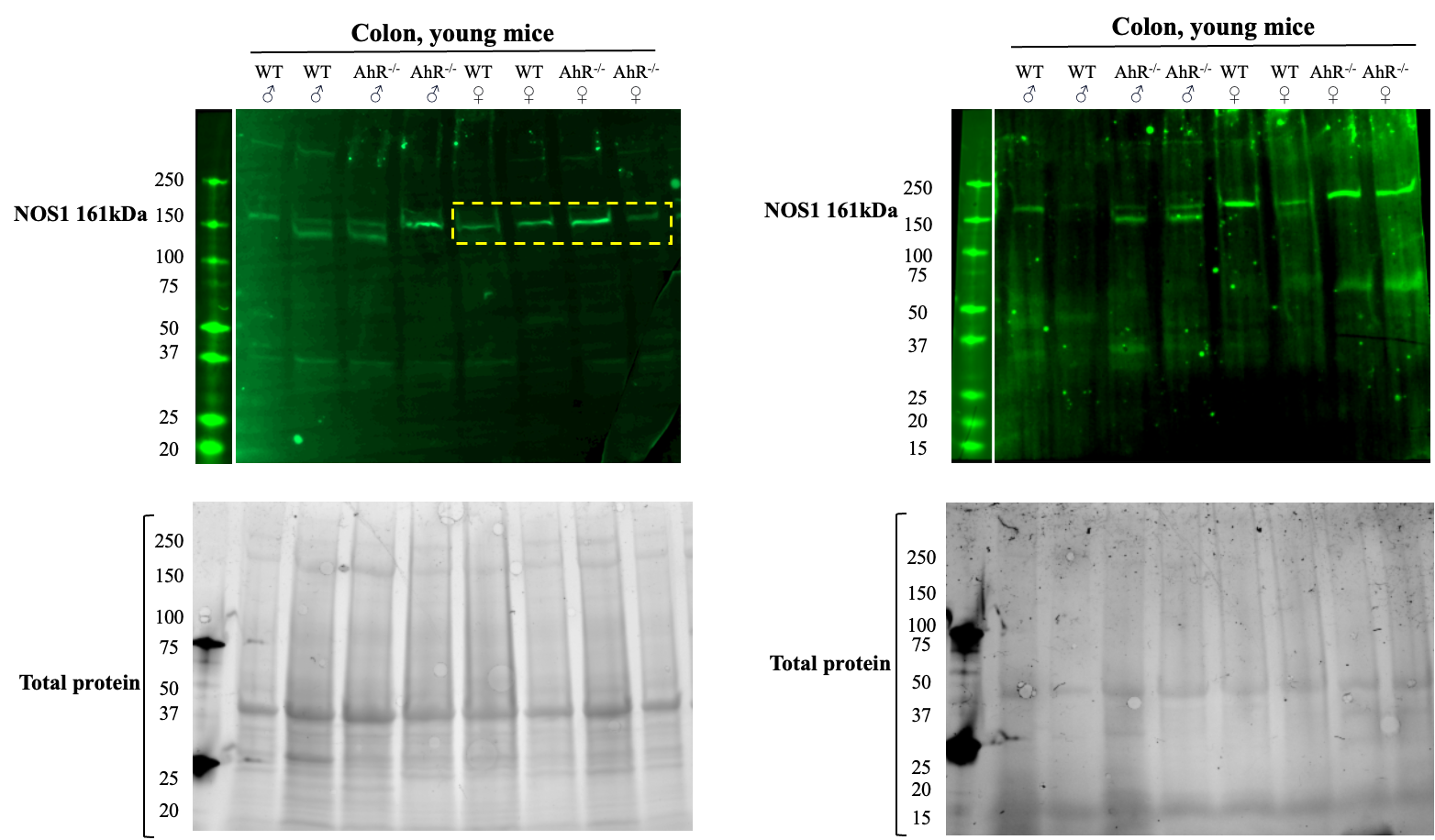


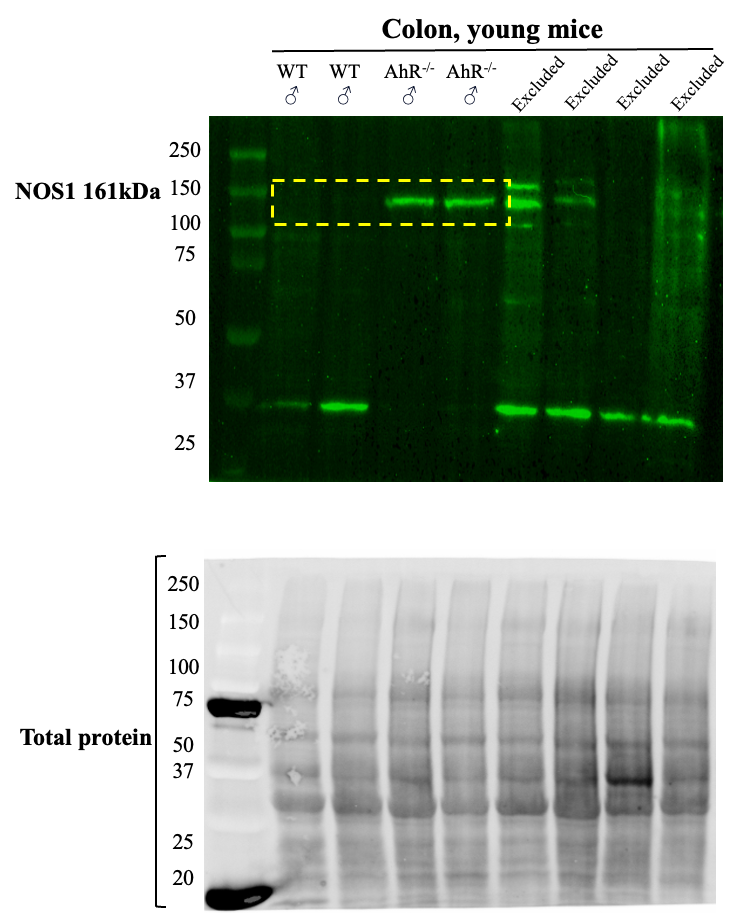


Complete Blots for figure 14B


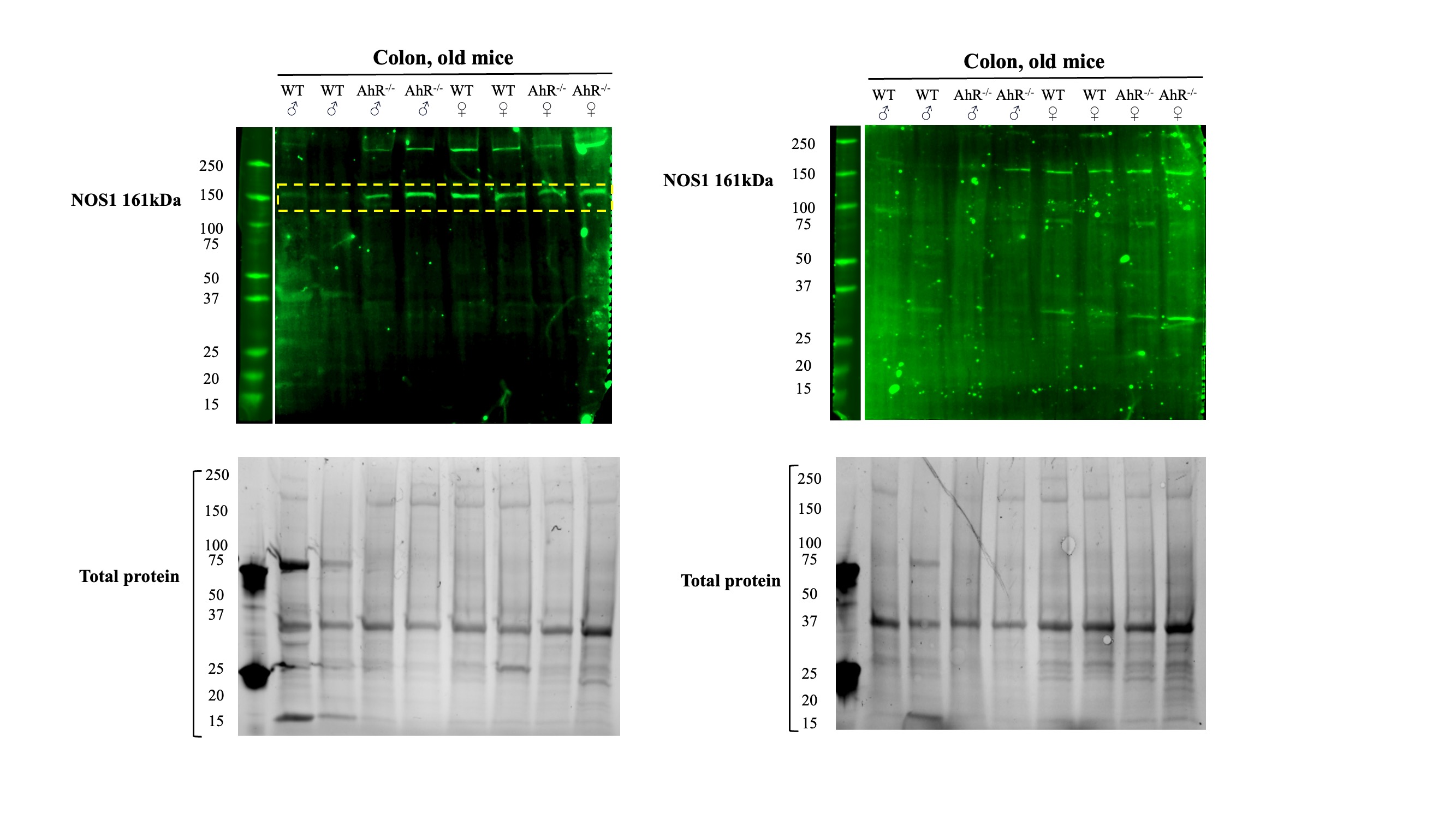

Supplement: Supplementary file 1 [file 13105_2026_1208_MOESM1_ESM.docx]
