# Supplementary figures and images for: Aryl hydrocarbon receptor deficiency leads to sex- and age-dependent colonic dysmotility in mice
Source: J Physiol Biochem. 2026 Jul 16;82(1):71. doi: 10.1007/s13105-026-01208-9 (PMC13375827; doi:10.1007/s13105-026-01208-9)

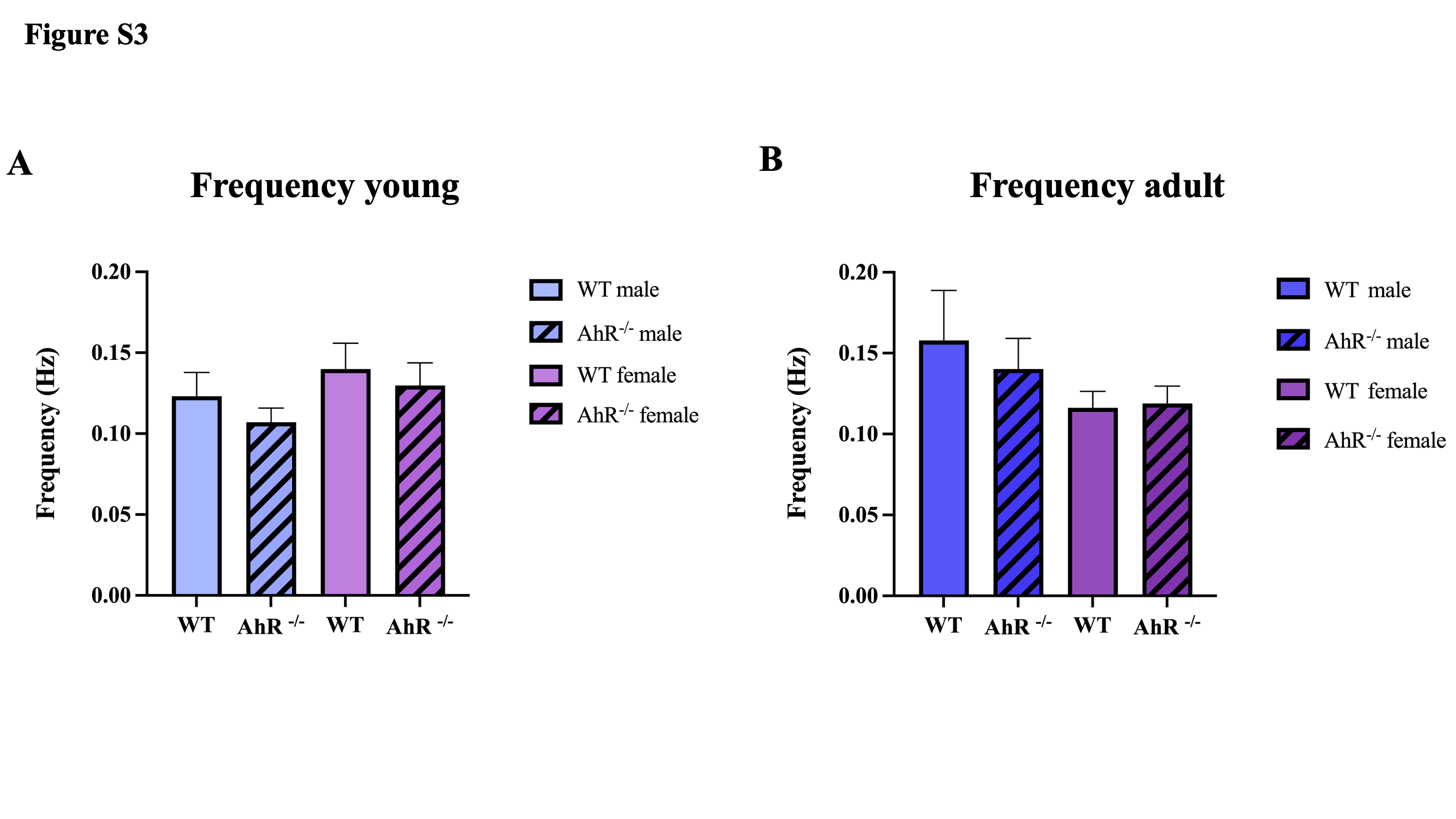

Supplement: Supplementary file 2 — Supplementary Figure S1. Representative images of 24-h fecal output from young male and female in WT and AhR-/- mice under standardized imaging conditions. Supplementary Figure S2. Representative images of 24-h fecal output from adult male and female in WT and AhR-/- mice under standardized imaging conditions. Supplementary Figure S3. Spontaneous peristalsis. Frequency (Hz) was measured in 1-cm-long colon segments of WT and AhR-/- male and female mice in young (A) and adult (B) groups. Data are expressed as mean ± SEM and were analyzed using two-way ANOVA followed by a Bonferroni post hoc test (n = 5 – 15 per group). [file 13105_2026_1208_MOESM2_ESM.zip › Supplementary 3.tiff]

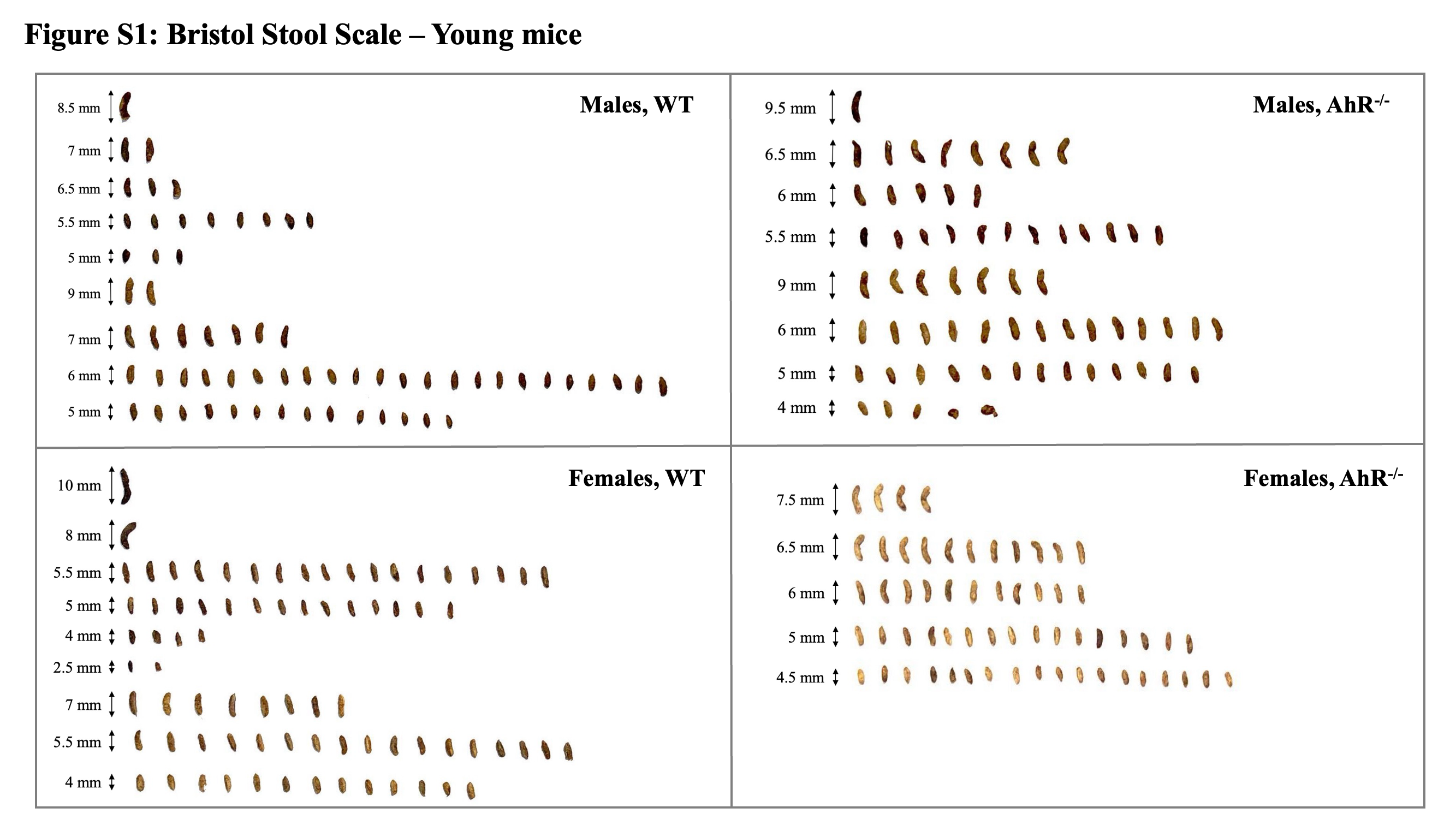

Supplement: Supplementary file 2 — Supplementary Figure S1. Representative images of 24-h fecal output from young male and female in WT and AhR-/- mice under standardized imaging conditions. Supplementary Figure S2. Representative images of 24-h fecal output from adult male and female in WT and AhR-/- mice under standardized imaging conditions. Supplementary Figure S3. Spontaneous peristalsis. Frequency (Hz) was measured in 1-cm-long colon segments of WT and AhR-/- male and female mice in young (A) and adult (B) groups. Data are expressed as mean ± SEM and were analyzed using two-way ANOVA followed by a Bonferroni post hoc test (n = 5 – 15 per group). [file 13105_2026_1208_MOESM2_ESM.zip › Supplementary 1.tiff]

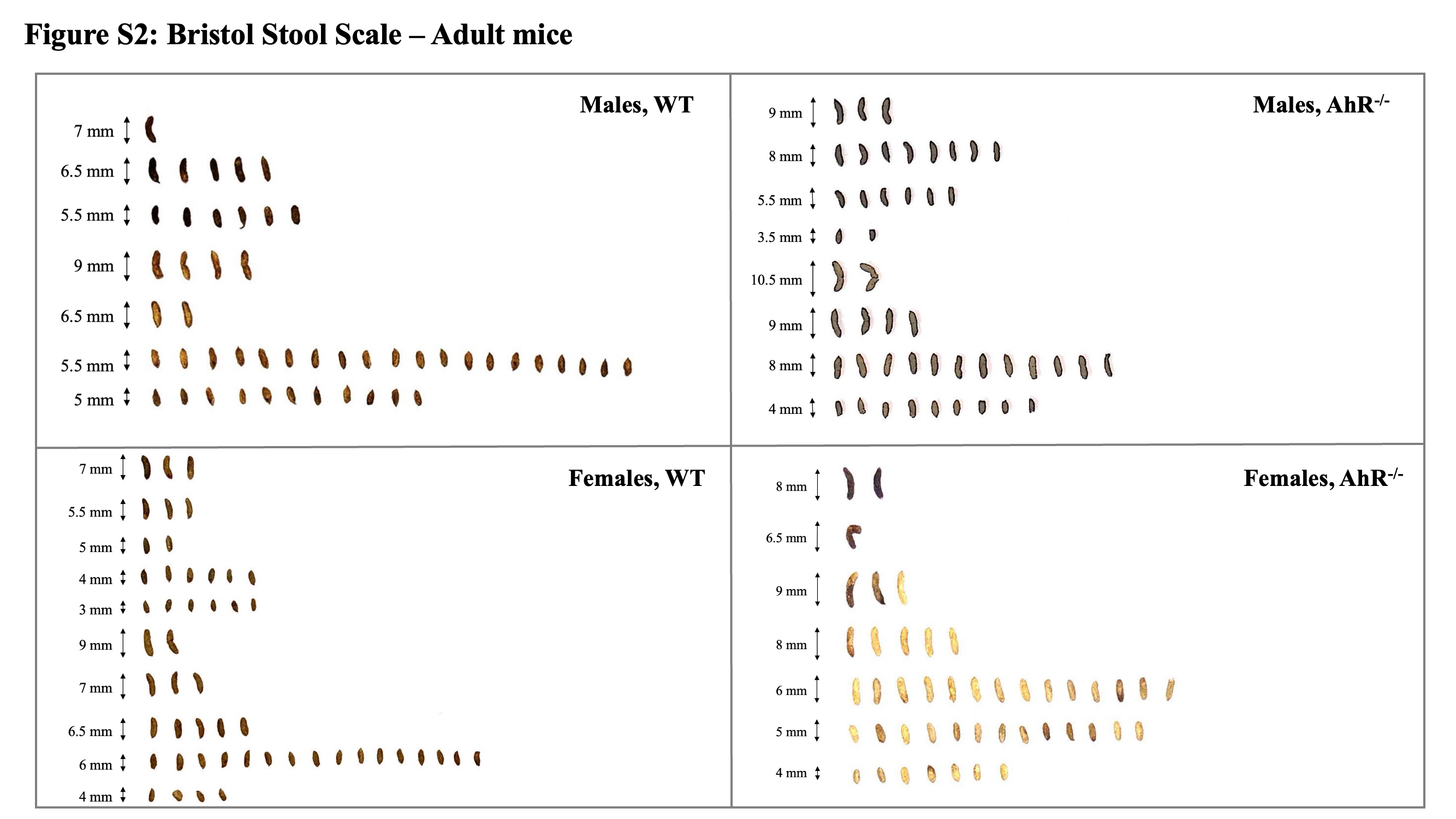

Supplement: Supplementary file 2 — Supplementary Figure S1. Representative images of 24-h fecal output from young male and female in WT and AhR-/- mice under standardized imaging conditions. Supplementary Figure S2. Representative images of 24-h fecal output from adult male and female in WT and AhR-/- mice under standardized imaging conditions. Supplementary Figure S3. Spontaneous peristalsis. Frequency (Hz) was measured in 1-cm-long colon segments of WT and AhR-/- male and female mice in young (A) and adult (B) groups. Data are expressed as mean ± SEM and were analyzed using two-way ANOVA followed by a Bonferroni post hoc test (n = 5 – 15 per group). [file 13105_2026_1208_MOESM2_ESM.zip › Supplementary 2.tiff]
